# A new cobalt(ii) *meso*-porphyrin: synthesis, characterization, electric properties and application in the catalytic degradation of dyes†

**DOI:** 10.1039/d0ra08786f

**Published:** 2020-12-18

**Authors:** Nesrine Amiri, Mouhieddinne Guergueb, Maged S. Al-Fakeh, Marwa Bourguiba, Habib Nasri

**Affiliations:** Laboratory of Physico-Chemistry of Materials, University of Monastir Avenue of the Environment 5019 Monastir Tunisia nesamiri@gmail.com +216 28256432; Department of Chemistry, Faculty of Science, Qassim University Saudi Arabia; Applied Mechanics and Systems Research Laboratory (LASMAP-EPT), Polytechnic School, University of Carthage La Marsa Tunisia; Faculty of Science of Tunis, University of Tunis El Manar Tunisia

## Abstract

In this work, a new porphyrin, 5,10,15,20-tetrakis{4-[((4-methoxyphenyl)acetyl)oxy]phenyl}porphyrin (H_2_TMAPP) (1), and its cobalt complex [Co^II^(TMAPP)] (2) were synthesized in good and quantitative yields, respectively. The chemical structures of these synthesized compounds were confirmed by FT-IR, ^1^H NMR, MS, UV-visible, and fluorescence spectroscopy. Their photophysical properties, namely their molar extinction coefficients (*∑*), fluorescence quantum yields (*Φ*_f_) and lifetimes (*τ*_f_), were determined and compared with those of *meso*-tetraphenylporphyrin. Furthermore, their electrochemical behaviours were examined using cyclic voltammetry (CV). Dielectric properties such as the conductivity (*σ*) and the real (*M*′) and imaginary (*M*′′) parts of the dielectric modulus were investigated as a function of temperature and frequency. The impedance analysis was carried out using Cole–Cole plots to elucidate the electrical conduction mechanism. The catalytic power and the adsorption properties of the prepared compounds were studied for methylene blue (MB) and crystal violet (CV) degradation. The results reveal that the studied compound [Co^II^(TMAPP)] can be used as a catalyst for the decolourisation of dyes in the presence of H_2_O_2_.

## Abstract 2

In this work, a new porphyrin, 5,10,15,20-tetrakis{4-[((4-methoxyphenyl)acetyl)oxy]phenyl}porphyrin (H_2_TMAPP) (1), and its cobalt complex [Co^II^(TMAPP)] (2) were synthesized in good and quantitative yields, respectively.

## Graphical abstract

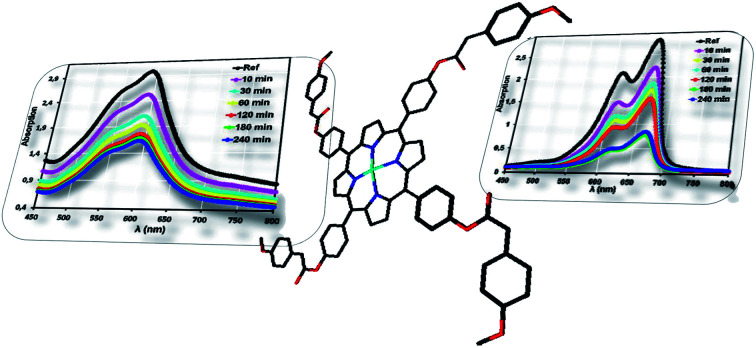

## 1. Introduction

**Scheme 1:**
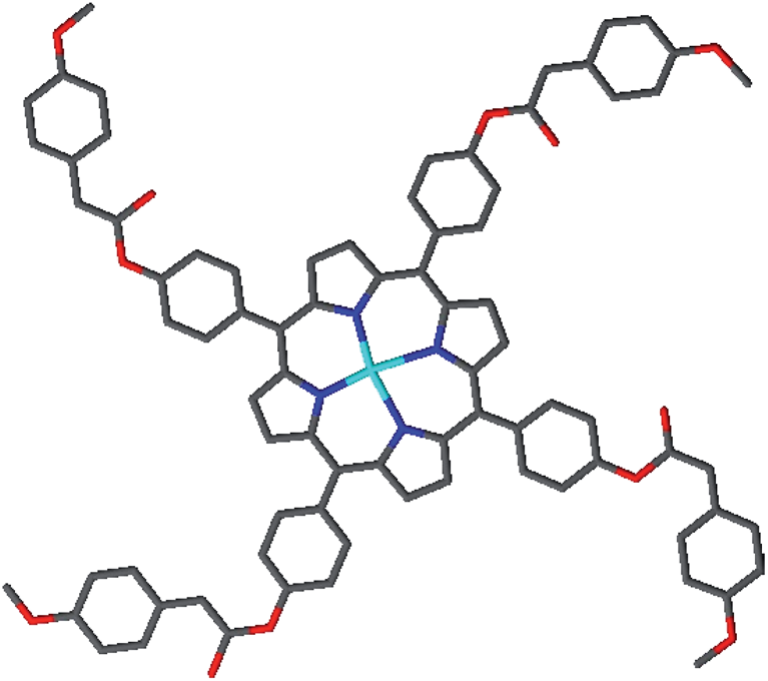
Molecular structure of *meso*-tetrakis{4-[((4-methoxyphenyl)acetyl)oxy]phenyl} porphyrinato cobalt(ii) (2) (drawing of the molecule).

**Scheme 2:**
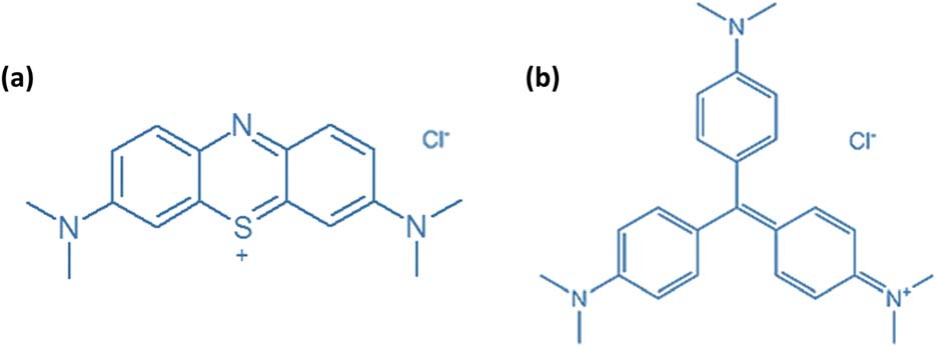
Molecular structures of (a) methylene blue (MB) and (b) crystal violet (CV).

Porphyrin, which is a large, widely conjugated cyclic planar molecule, is attracting the attention of many researchers in various fields due to its remarkable photochemical, electrochemical, and biochemical properties.^1^ The synthesis and functionalization of this macrocycle have long been of interest because of the potential of porphyrin derivatives in diverse fields, such as catalysis, photocatalysis, molecular wires,^2–4^ sensitive reagents in photodynamic therapy (PDT) for tumors and cancers,^5^ functional dyes and pigments,^6^ solar cells,^7^ energy transfer, and light-harvesting.^8–11^ The substitution of suitable organic groups on the periphery of porphyrin rings as well as the coordination of the metal in the center of the porphyrin ring permit the design of new synthetic porphyrins. Substitution at the *meso* positions of highly aromatic and bulky groups, such as naphthyl, phenanthryl, or pyrenyl porphyrins, has been reported, which alters its aromatic character. The development of ester functions with porphyrins has attracted much attention due to their wide range of applications.^12–14^ For example, Carminati *et al.*^13^ described the preparation of an iron porphyrin substituted at the *meso* position by amino ester groups. These complexes are emerging as candidates in catalysis measurements. Meden F. Isaac *et al.*^14^ described the preparation of novel porphyrin dimers bearing ester groups in the *meso* position. These compounds have potential applications in binary cancer therapies. In another example, Olycen Oviedo *et al.*^12^ synthesized *meso*-tetra-(4-benzoate-9-phenanthryl)-porphyrin and its Zn and Cu complexes, which have been studied both in the photosensitized oxidation of phenols and photoinduced antibacterial efficiency. In addition, the development of methoxyphenyl functions with porphyrins has been the subject of particular attention due to their catalytic activities, *i.e.* they have been used as catalysts for biomimetic oxidation^15,16^ and for the degradation of green dye.^17^ Recently, our research group managed to design and characterize new metalloporphyrin-based materials.^18–20^ In this paper, and in continuation of our work on the functionalization of *meso* tetra-arylporphyrins, we have synthesised a new porphyrin, H_2_TMAPP (1) (TMAPP = 5,10,15,20-tetrakis{4-[((4-methoxyphenyl)acetyl)oxy]phenyl}porphyrin), and its cobalt complex [Co^II^(TMAPP)] (2), which are substituted at the *meso* position by ester groups comprising two benzene rings plus methoxy functions (Scheme 1). With this aim, our compounds have been characterized by infrared, proton nuclear magnetic resonance spectroscopy, mass spectrometry, and elemental analysis. We also report on their optical and electrochemical properties. Therefore, in this work, attention has been focused on the investigation of the conductivity (*σ*) and the real (*M*′) and imaginary (*M*′′) parts of the dielectric modulus as a function of frequency and temperature of Co(ii)-porphyrin to obtain the maximum amount of information from the experimental data. The impedance (*Z*) was also investigated. Further, their dye adsorption properties and catalytic degradation using methylene blue (MB) and crystal violet (CV) dyes (Scheme 2) were evaluated.

## 2. Experimental

### 2.1. General

All solvents and reagents were purchased from Sigma Aldrich or ACROS ORGANICS. Used solvents were purified using the available literature methods.^21^ Silica gel 150 (35–70 μm particle size, Davisil) was used for final purification of the products. Double distilled water was used in the experiments. The starting material, the aldehyde 4-formylphenyl(4-methoxyphenyl) acetate, was prepared as previously described.^22^ The macrocycle of H_2_TMAPP was synthesized and purified according to the procedures described by Lindsey *et al.*,^23^ and cobalt was inserted into the porphyrin molecule by the dimethylformamide method^24,25^ (Scheme S1†).

### 2.2. Synthesis

#### Synthesis of 4-formylphenyl (4-methoxyphenyl)acetate

4-Methoxyphenylacetic acid (6 g, 0.036 mol), 4-hydroxybenzaldehyde (4.4 g, 0.036 mol), and dimethylaminopyridine (DMAP, 0.5 g, 0.0036 mol.) were dissolved in dichloromethane (20 mL) at 0 °C. To this solution, *N*,*N*′-dicyclohexylcarbodiimide (DCC) (7.5 g, 0.036 mol) in dichloromethane (25 mL) was added dropwise, and the mixture was stirred at 0 °C and then at room temperature for 12 h. Upon completion of the reaction, the mixture was filtered, and dichloromethane was removed by rotary evaporation. The solution was poured into water, and the solid was collected by filtration, washed with water followed by hexanes, and dried under vacuum to afford a pale-yellow powder (9 g, yield 92.5%). Anal. Calcd. For C_16_H_14_O_4_ (270.28): C 71.04, H 5.22%; found C 71.26, H 5.34%. ^1^H NMR (CDCl_3_, 300 MHz): *δ* = 9.98 (s, 1H), 7.95 (d, *J* = 6.4 Hz, 2H), 7.32 (d, *J* = 8.7 Hz, 4H), 6.92 (d, *J* = 7.7 Hz, 2H), 3.90 (s, 3H), 3.75 (s, 2H) ppm. ^13^C NMR (CDCl_3_, 300 MHz): *δ* = 191.73 (C=O of CHO), 163.68 (C=O of ester), 54.97 (C–O of methoxy), 158.69, 155.04, 131.39, 130.72, 122.37 ppm.

#### Synthesis of the *meso*-tetrakis{4-[((4-methoxyphenyl)acetyl)oxy]phenyl}porphyrin (H_2_TMAPP) (1)

4-Formylphenyl (4-methoxyphenyl)acetate (500 mg, 1.85 mmol), and pyrrole (127 μL, 1.85 mmol) were added to distilled chloroform (300 mL) in a double necked round bottom flask under argon and shielded from light. Boron trifluoride diethyl etherate, BF_3_·OEt_2_ (192.4 μL) was added, and the reaction was maintained at room temperature for two hours. Two pipettes of triethylamine and 0.75 equivalents of *p*-chloranil (179.2 mg, 1.66 mmol) were added, and the solution was heated to reflux (light protection was removed). After 1 hour, the obtained solution was cooled to room temperature. The solvent was evaporated and the residue was filtered over silica with CHCl_3_/hexane (1 : 9). The expected compound was obtained as a purple solid (yield 69%). ^1^H NMR [DMSO-d_6_, 300 MHz] *δ*(ppm): 8.83 (s, 8H, H_β_-pyrrol), 8.23 (d, *J* = 7.4 Hz, 8H), 7.56 (d, *J* = 7.5 Hz, 8H), 7.44 (d, *J* = 7.5 Hz, 8H), 7.02 (d, *J* = 7.5 Hz, 8H), 4.06 (s, 8H), 3.81 (s, 12H), −2.88 (s, 2H, hpyrrol). UV/vis [*λ*_max_ (nm) in CH_2_Cl_2_, (log *ε*)]: 425 (5.92), 522 (5.67), 550 (4.32), 597 (4.15), 653 (3.98). MS [ESI]: *m*/*z* calcd for C_80_H_62_N_4_O_12_: 1271.36 found: 1272.36. Anal. calcd for C_80_H_62_N_4_O_12_: C 75.51, H 4.87, N 4.40%; found: C 75.77, H 4.56, N 4.42%. FTR-IR cm^−1^: 3323 (*ν*_NH_ porphyrin), 2897 (*ν*_CH_ porphyrin), 1736 (*ν*_C=O_ ester), 1285 (*ν*_C–O_ ester), 1225 (*ν*_O–C_ methoxy), 987 (*δ*_CCH_ porphyrin).

#### Synthesis of the *meso*-tetrakis{4-[((4-methoxyphenyl)acetyl)oxy]phenyl} porphyrinato cobalt(ii) complex [Co^II^(TMAPP)] (2)

H_2_TMAPP (0.4 g, 0.314 mmol) was dissolved in DMF (150 mL). The solution was heated under reflux with magnetic stirring. Upon dissolution of the H_2_TMAPP, CoCl_2_·6H_2_O (1 g, 4.2 mmol) was added. The reaction mixture was stirred for 2 hours. Thin-layer chromatography (alumina, using CH_2_Cl_2_ as eluant) indicated no free base porphyrins at this point. After that, the solution was cooled to 50–60 °C, and H_2_O (50 mL) was added to it. The obtained solid was filtered and washed with hexane. The resulting product was vacuum-dried to afford 66% yield of [Co^II^(TMAPP)]. ^1^H NMR [DMSO-d_6_, 300 MHz] *δ*(ppm): 15.69 (s, 8H, H_β_-pyrrol), 9.25 (d, *J* = 7.5 Hz, 8H), 8.81 (d, *J* = 7.8 Hz, 8H), 8.49 (d, *J* = 7.7 Hz, 8H), 7.75 (d, *J* = 7.5 Hz, 8H), 4.12 (s, 8H), 3.89 (s, 12H). UV/vis [*λ*_max_ (nm) in CH_2_Cl_2_, (log *ε*)]: 415 (5.85), 539 (5.49). MS [ESI]: *m*/*z* calcd for C_80_H_60_CoN_4_O_12_: 1328.28, found: 1329.29. Anal. calcd for C_80_H_60_CoN_4_O_12_: C 72.27, H 4.52, N 4.22%; found: C 72.34, H 4.23, N 4.07%. FTR-IR cm^−1^: 2923 (*ν*_CH_ porphyrin), 1737 (*ν*_C=O_ ester), 1260 (*ν*_C–O_ ester), 1226 (*ν*_O–C_ methoxy), 993 (*δ*_CCH_ porphyrin).

### 2.3. Instrumentation

#### IR and ^1^H NMR spectroscopy

IR and ^1^H NMR spectra were recorded on a Nicolet Impact 410 and a Bruker AVANCE (300 MHz) spectrometer, respectively. ^1^H NMR measurements were carried out at room temperature.

#### Mass spectral studies

A Thermo Scientific “Q Exactive” mass spectrometer was operated under electrospray ionization (ESI) in positive mode with the following settings: accelerating voltage 20 kV, grid voltage 62% of the accelerating voltage, extraction delay time of 100 ns.

#### Optical measurements

UV-vis spectra of H_2_TMAPP and [Co^II^(TMAPP)] were measured with a Varian Cary 5000 spectrophotometer in CH_2_Cl_2_ solution at concentrations of *ca.* 10^−6^ M. The two samples were characterized to see the displacement of the Soret band. From these spectra, the molar extinction coefficient, *ε*, of the Soret band and secondary bands were calculated. A Varian Cary Eclipse luminescence spectrofluorometer was used to obtain emission spectra in dichloromethane at room temperature, and the fluorescence quantum yield (*Φ*_f_) was measured using the following equation:^26,27^

1 *Φ*_f_/*Φ*_r_ = (*A*_r_/*A*_f_)(*F*_f_/*F*_r_)(*n*_f_^2^/*n*_r_^2^)

In this relation, *Φ*_r_, *A*_r_, *F*_r_ and *n*_r_ are the fluorescence quantum efficiency, absorbance at excitation wavelength, emission integration area, and refractive index for the reference, while *Φ*_f_, *A*_f_, *F*_f_ and *n*_f_ are those of the tested porphyrinic species. [Zn^II^(TPP)] was used as reference with the standard quantum yield of *Φ*_f_ = 0.03.^28^

#### Electrochemical analysis

Cyclic voltammetry (CV) experiments were performed with a CH-660B potentiostat (CH Instruments). All analytical experiments were conducted at room temperature under an argon atmosphere (argon stream) in a standard one-compartment, three-electrode electrochemical cell. Tetra-*n*-butylammonium perchlorate (TBAP) was used as the supporting electrolyte (0.2 M) in dichloromethane previously distilled over calcium hydride under argon. An automatic ohmic drop compensation procedure was systematically implemented before the CV data were recorded with electrolytic solutions containing the studied compounds at concentrations of *ca.* 10^−3^ M. CH Instruments vitreous carbon (*Φ* = 3 mm) working electrodes were polished with 1 μm diamond paste before each recording. The Ag/AgNO_3_ 0.01 M (TBAP 0.2 in CH_2_Cl_2_) redox couple was used as the reference electrode. The potential of the ferrocene/ferrocenium redox couple was used as an internal reference (86 mV *vs.* Ag/AgNO_3_ under our experimental conditions). For comparison with previously published data, all potentials given in the text and in Table S2† have been converted to values relative to the saturated calomel electrode (SCE) by using the following relationship: *E*(SCE) = *E*(Ag/AgNO_3_) + 298 mV.^29^

#### Adsorption experiments

The adsorption of methylene blue (MB) and crystal violet (CV) (Scheme 2) in the presence of H_2_TMAPP and [Co^II^(TMAPP)] was carried out in batch mode with constant agitation. Kinetic experiments were studied by stirring a series of flasks enclosing 5 mL of methylene blue solution with 3 mg of adsorbent with the same stirring speed. After the required time was reached, the supernatant liquid was filtered. The concentration of methylene blue was measured at 664 nm and that of crystal violet was measured at 591 nm.

#### Catalytic degradation of methylene blue and crystal violet

Two different organic dye pollutants, namely, methylene blue (MB) and crystal violet (CV), were chosen to investigate the catalytic activity of [Co^II^(TMAPP)] (2). In a typical experiment, to a 10 mL aqueous solution of dye (30 mg L^−1^) was added 1 mL of H_2_O_2_ (30 wt%) and 3 mg of [Co^II^(TMAPP)] (2). Then, a preferred amount of catalyst was added to this mixture at an agitation speed of 150 rpm. The reaction solution was pipetted into a quartz cell and its absorption spectrum was measured for different reaction times using an UV-visible spectrophotometer. Blank experiments were also conducted to confirm that the reactions did not proceed with catalyst in the absence of H_2_O_2_ or without catalyst in the presence of H_2_O_2_.

## 3. Results and discussion

### 3.1. IR and ^1^H NMR spectroscopies

In order to gain more insights into the structures of the porphyrin and metalloporphyrin, detailed IR and ^1^H NMR studies were performed. Representative solid-state infrared spectra are shown in Fig. S1 and S2.† The spectra of the free porphyrin H_2_TMAPP and metalloporphyrin [Co^II^(TMAPP)] were very similar, and only minor frequency shifts were observed. The only obvious difference arose from the disappearance of the N–H (3323 cm^−1^) vibration frequency of free base porphyrin, indicating the formation of cobalt(ii) porphyrin compound (Fig. S2†). Our compounds are in general agreement with those recently reported for a number of porphyrins.^19,28^ The 2897 and 2923 cm^−1^ peaks are associated with antisymmetric and symmetric alkyl C–H vibrations. The strongest absorption peaks in the spectra at 1736 and 1260 cm^−1^ can be associated with the ester group stretch. The bands observed around 1000 cm^−1^ are assigned to *δ*(CCH) vibration modes for the *meso*-porphyrin. The ^1^H NMR spectra of 1 and its Co(ii) derivative 2 were recorded in deuterated chloroform at 298 K (Fig. S3 and S4†). The free base porphyrin shows characteristic inner amino proton (NH) signals at −2.88 ppm which are absent in metalloporphyrin. The β-pyrrole protons resonate as a singlet at 8.83 ppm, and the aromatic protons of the *meso*-phenyl rings resonate in the range of 8.24–7.00 ppm, which corresponds to a classical case for all *meso*-porphyrins. The methyl and methoxy protons of *para*-methoxy phenolate appear at 4.06 and 3.81 ppm, respectively. [Co^II^(TMAPP)] in CDCl_3_ has paramagnetic properties, as shown by the chemical shifts and the broadened singlets of the H_β_-pyrrolic protons (at 15.69 ppm).^30^ This property is due to the 3d configuration of cobalt(ii).

### 3.2. Mass spectral studies

Mass spectroscopy is widely used in the study of porphyrins to determine the molecular weights of porphyrins and metalloporphyrins by ESI-mass spectroscopic techniques in the positive ion mode. The molecular spectra of compounds 1 and 2 (Fig. S5 and S6†) are best recorded at the lowest possible temperature. These mass spectra show the molecular ion peaks of these compounds, which are in good agreement with the structures suggested by elemental analysis and spectral studies. The protonated molecular ion peaks, [M] or [M + H], of H_2_TMAPP were observed at 1271.36 (Fig. S5†). In the MALDITOF mass spectrum of the cobalt porphyrin, a molecular ion peak was observed at 1328.29 (Fig. S6†). These mass values overlapped with the theoretically calculated mass values for the [M] or [M + H] of the two compounds.

### 3.3. Optical properties

#### UV-vis absorption spectroscopy

**Fig. 1:**
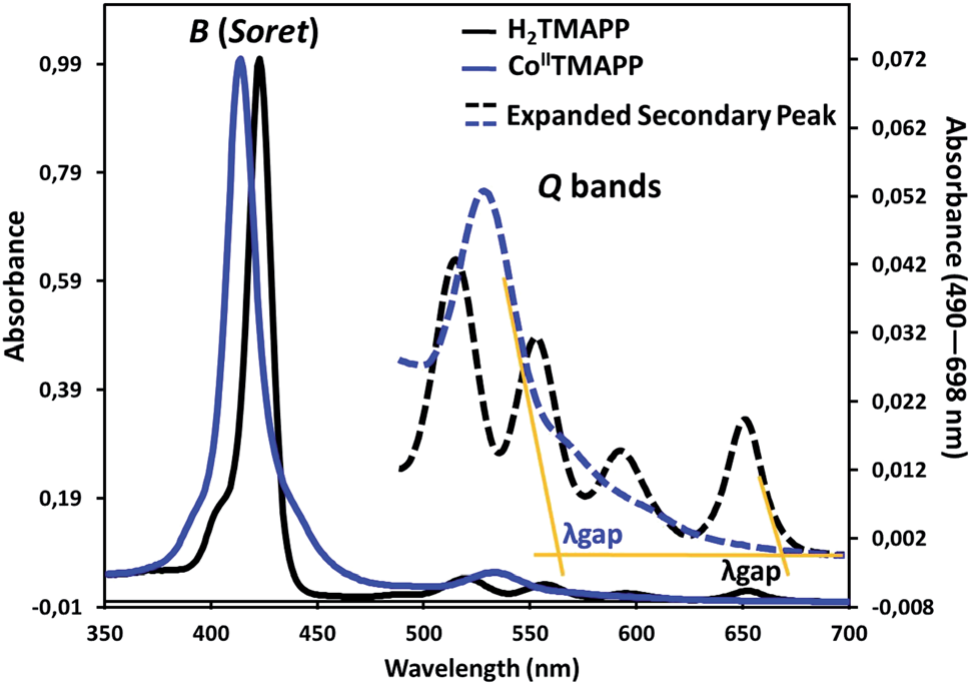
UV-vis absorption spectra of H_2_TMAPP and [Co^II^(TMAPP)] in CH_2_Cl_2_ solutions at concentrations around 10^−6^ mol L^−1^.

Fig. 1 shows the UV-visible spectrum of the demetallated macrocycle H_2_TMAPP compared with the response of the metallated macrocycle [Co^II^(TMAPP)]. The ground state electronic absorption spectra of the porphyrins are characterized by an intense band called the Soret or B band at around 400 nm. The Soret band of H_2_TMAPP was observed at 425 nm (log *ε* = 5.92) (Table S1†), while the four Q bands, decreasing in intensity, were at 522 nm (log *ε* = 5.67), 550 nm (log *ε* = 4.32), 597 nm (log *ε* = 4.15) and 653 nm (log *ε* = 3.98) (Fig. 1). The Q bands decreased in intensity in the following order: Q_*y*_(1,0) > Q_*y*_(0,0) > Q_*x*_(1,0) > Q_*x*_(0,0). These absorption bands are characteristic for porphyrins and are due to the a_1u_(π) − e_g_(π*) and a_2u_(π) − e_g_(π*) transitions for the Soret and Q bands, respectively.^31,32^ The four Q-bands of the metal-free precursor collapsed into one Q band following metalation, confirming the successful insertion of cobalt as a central metal. The Q band of [Co^II^(TMAPP)] was observed at 534 nm (log *ε* = 5.49), while the Soret band was observed at 415 nm (log *ε* = 5.85), as shown in Fig. 1 (Table S1†), showing that there is a 10 nm blue shift for [Co^II^(TMAPP)] compared to H_2_TMAPP. This blue shift is a result of the insertion of the central metal and generates an increase of the symmetry (from *D*_2h_ to *D*_4h_), and the introduction of a heavy metal such as cobalt could result in an increase in the degree of perturbation and electron delocalisation within the porphyrin macrocycle.

The optical band gap (*E*_g-op_), which corresponds to the energy difference between the levels of the HOMO and LUMO, was obtained from the UV-visible spectra. This energy was calculated from the value of the tangent to the Q(0,0) absorption band (*λ*_gap_). The *E*_g-op_ values were 1.85 eV (*λ*_gap_ = 670 nm) for H_2_TMAPP and 2.18 eV (*λ*_gap_ = 568 nm) for [Co^II^(TMAPP)], which are in the normal range of *meso*-porphyrins and magnesium metalloporphyrins.^19,33,34^

#### Steady-state emission spectroscopy

**Fig. 2:**
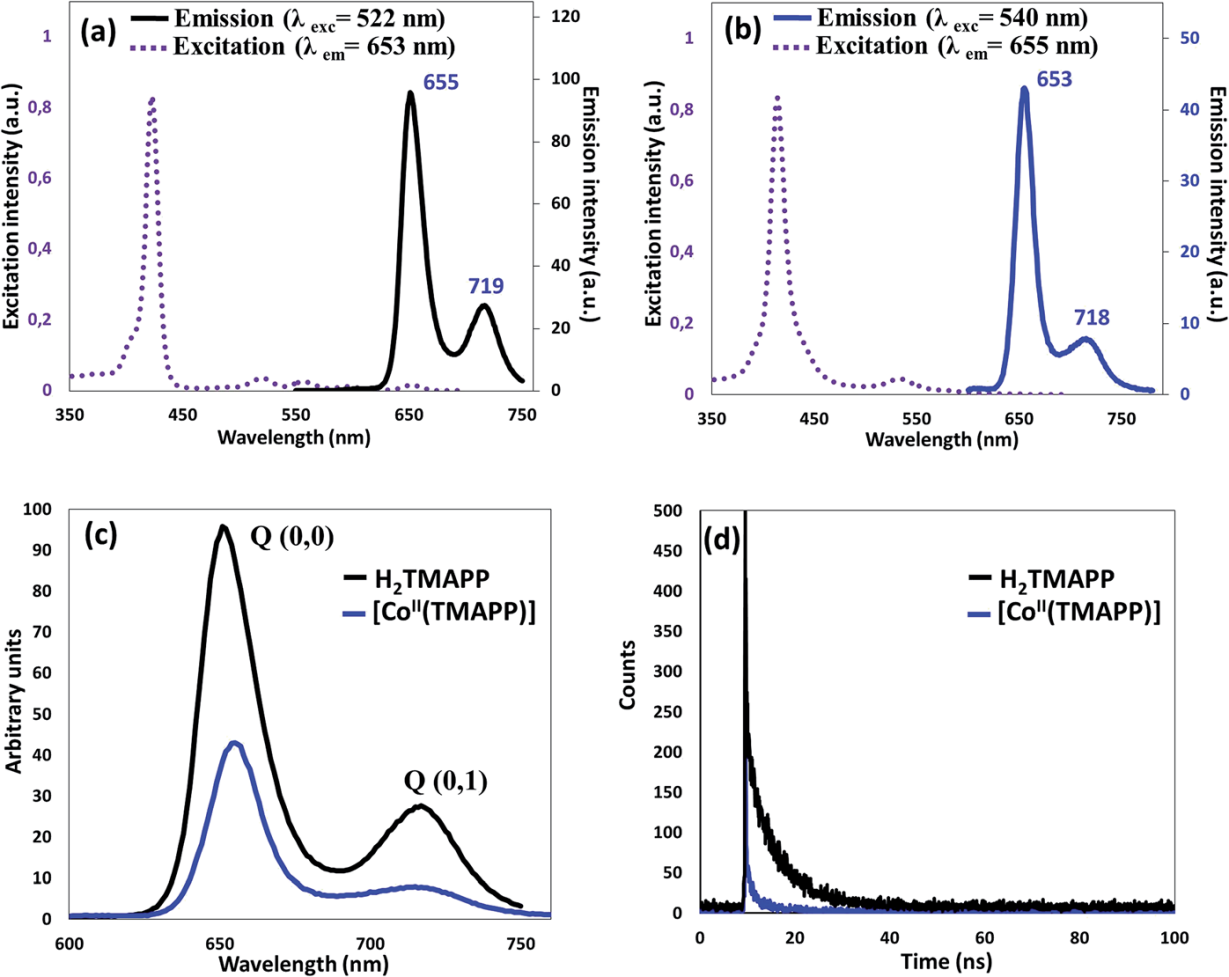
(a) Excitation and emission spectra of H_2_TMAPP, (b) excitation and emission spectra of [Co^II^(TMAPP)], (c) emission spectrum of H_2_TMAPP and [Co^II^(TMAPP)] as 10^−6^ mol L^−1^ solutions in dichloromethane, (d) fluorescence decay profiles of (1) and (2).

The room temperature fluorescence spectra in dichloromethane (10^−6^ mol L^−1^) are shown in Fig. 2. The fluorescence emission is attributed to the transition from the excited singlet state S_1_/S_2_ to the ground state S_0_ (S_2_ → S_0_, S_1_ → S_0_). The fluorescence of the B (Soret) band is attributed to the transition from the second excited singlet state S_2_ to the ground state S_0_, S_2_ → S_0_, and the Soret fluorescence is about 2 orders of magnitude weaker than that of the S_1_ → S_0_ transition of the Q band emission. Upon excitation at 522 nm, the free base porphyrin displayed two emission peaks of S_1_ → S_0_, centered at 655 nm and 719 nm (Fig. 2a). The spectrum of the metalloporphyrin [Co^II^(TMAPP)] showed a very weak fluorescence band, and when excited at 540 nm, it gave two split emission bands at 653 nm (S_1_ [Q(0,0)] → S_0_) and at 718 nm (S_1_ [Q(0,1)] → S_0_) (Fig. 2b). It is noteworthy that the *λ*_max_ values of the Q(0,0) and Q(0,1) bands of these compounds are practically the same; the bands in the complex are similar to those in the free porphyrin but with a lower intensity (Fig. 2c). As previously reported, the decrease in the emission band intensity of compound 1 is probably due to the cobalt atom effect.^18,35–38^ In the region of 350–700 nm, the excitation spectra were approximately mirror images of the absorption spectra, indicating that they correspond to a similar electron transition process (Fig. 2a and b). The fluorescence quantum yields (*Φ*_f_) of the starting material H_2_TMAPP and [Co^II^(TMAPP)] were 0.050 and 0.028, respectively. The singlet excited-state lifetimes were measured by the single-photon counting technique, and the fluorescence decays of the starting material (1) and (2) species were fitted to single exponentials. The representative fluorescence decays (*τ*_f_) of these two derivatives are presented in Fig. 2d. As expected, the *τ*_f_ values of the *meso*-porphyrin free bases (*ca.* 5.93 ns) are much higher than those of [Co^II^(TMAPP)] (1.5 ns).

### 3.4. Electrochemical characterization

**Fig. 3:**
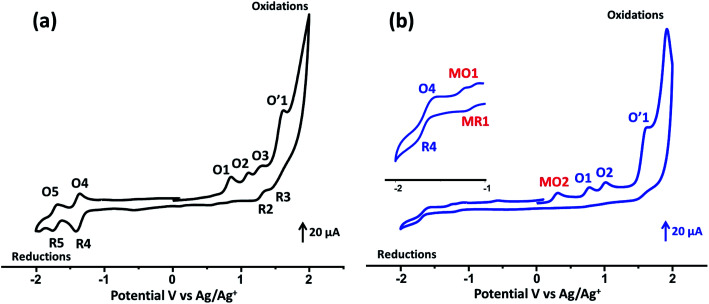
Cyclic voltammograms of (a) H_2_TMAPP and (b) [Co^II^(TMAPP)] (the inset shows an enlarged view). The solvent was CH_2_Cl_2_, and the concentration was *ca.* 10^−3^ M in 0.2 M TBAP, 100 mV s^−1^, vitreous carbon working electrode (*Ø* = 3 mm).

The cyclic voltammograms (CV) of the studied porphyrins in tetra-*n*-butylammonium perchlorate (TBAP) electrolyte (0.2 M) in the non-coordinating solvent CH_2_Cl_2_ under an argon atmosphere are displayed in Fig. 3, and all potential values are given in volts *versus* SCE. The free base H_2_TMAPP exhibited two reversible one-electron reduction waves and three reversible one-electron oxidation waves, which correspond to the reduction and the oxidation of the porphyrin ring, respectively (Fig. 3a). The values of the half-wave potentials were 1.15 (O1), 1.46 (O2, R2), and 1.66 (O3, R3) V for the first, second, and third oxidation waves, respectively. The *E*_1/2_ values of the first and second reduction waves were −1.08 (R4, O4) and −1.41 (R5, O5) V, respectively. The *E*_1/2_ values of H_2_TMAPP are close to those of *meso*-porphyrin (Table S2†).^17,39^ Concerning our Co(ii)-TMAPP derivative (2), it presents three one-electron reversible oxidations (Fig. 3b), where the first corresponds to the center metal [Co(ii)/Co(iii)] (MO2) oxidation followed by two ring oxidations, with an *E*_ap_ (*E*_ap_ = anodic peak potential) value of 0.61 V; this is in the range of 0.60–0.98 V, which has been shown in several solvents for cobalt metalloporphyrins (Table S2†).^17,40–42^ The reduction of the same [Co^II^(TMAPP)] species leads to reduction of the centre metal [Co(ii)/Co(i)] (with *E*_1/2_ = −0.93 V (MR1, MO1)) followed by a second ring cantered one-electron reduction at quite negative potentials (with *E*_1/2_ = −1.38 V (R4, O4)). Moreover, these two compounds exhibit an irreversible oxidation wave (O′1) with an *E*_ap_ value of 1.91 V, which can be attributed to oxidation of the phenyl rings of the *meso*-porphyrin.^43^

### 3.5. Complex impedance spectroscopy

#### Conductivity

**Fig. 4:**
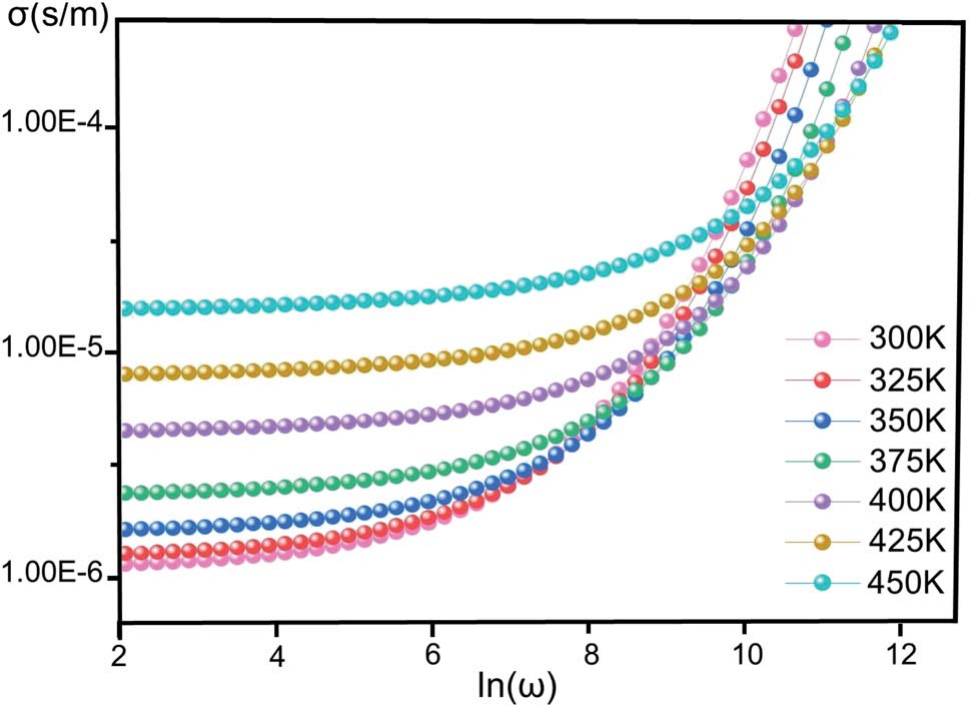
The conductivity (*σ*) *versus* the frequency of [Co^II^(TMAPP)] at various temperatures.

Fig. 4 illustrates the variation of *σ*(*ω*) *vs.* ln(*ω*) for [Co^II^(TMAPP)] in the temperature range of 300–450 K. It is clear that the frequency of *σ*(*ω*) remains constant at low frequency and after a certain frequency; it increases with the power law with increasing frequency. This behavior has also been observed in other organic compounds.^44–47^ This behaviour confirms the result found by the complex impedance. The *σ*(*ω*) can be represented dynamically by the universal response (Jonscher power law) relation:^48^

2 *σ*(*ω*,*T*) = *σ*_dc_(*T*) + *σ*_ac_(*ω*,*T*)

where *σ*_dc_ is related to direct conduction (independent of frequency while temperature dependent) and *σ*_ac_ is related to relaxation or polarization conductivity (frequency dependent and weakly temperature dependent). The polarization conductivity is described by the relation:^49^

3 *σ*_ac_(*ω*,*T*) = *Aω*^*s*^

where *A* is a pre-exponential factor which depends on the temperature, *ω* (=2π*f*) is the angular frequency and the exponent *s* represents the degree of interaction between mobile ions and the lattice.

**Fig. 5:**
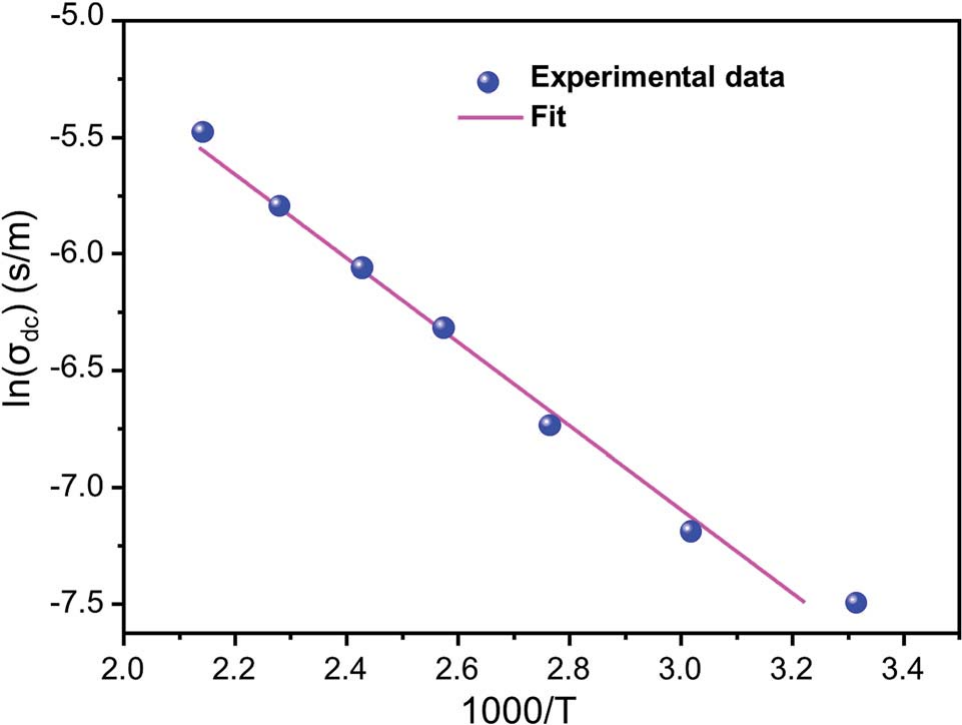
Variation of ln(*σ*_dc_) as a function of (1000/*T*).

Fig. 5 shows ln(*σ*_dc_) as a function of 1000/*T*. We note that ln(*σ*_dc_) decreases linearly with increasing reciprocal temperature. This indicates that the dc conductivity is a thermally activated process. The temperature dependence of *σ*_dc_ can be represented by the usual Arrhenius relation:^50,51^

4 *σ*_dc_ = *σ*_0_ exp(−Δ*E*_dc_/*k*_B_*T*)

where *σ*_0_ is a constant that denotes the pre-exponential factor, Δ*E*_dc_ is the activation energy for the conduction process, *k*_B_ is Boltzmann's constant and *T* is the absolute temperature. The linear fit to this graph yields the activation energy Δ*E*_as_ 0.326 eV.

#### Complex impedance

The complex impedance is represented by the equation

**Fig. 6:**
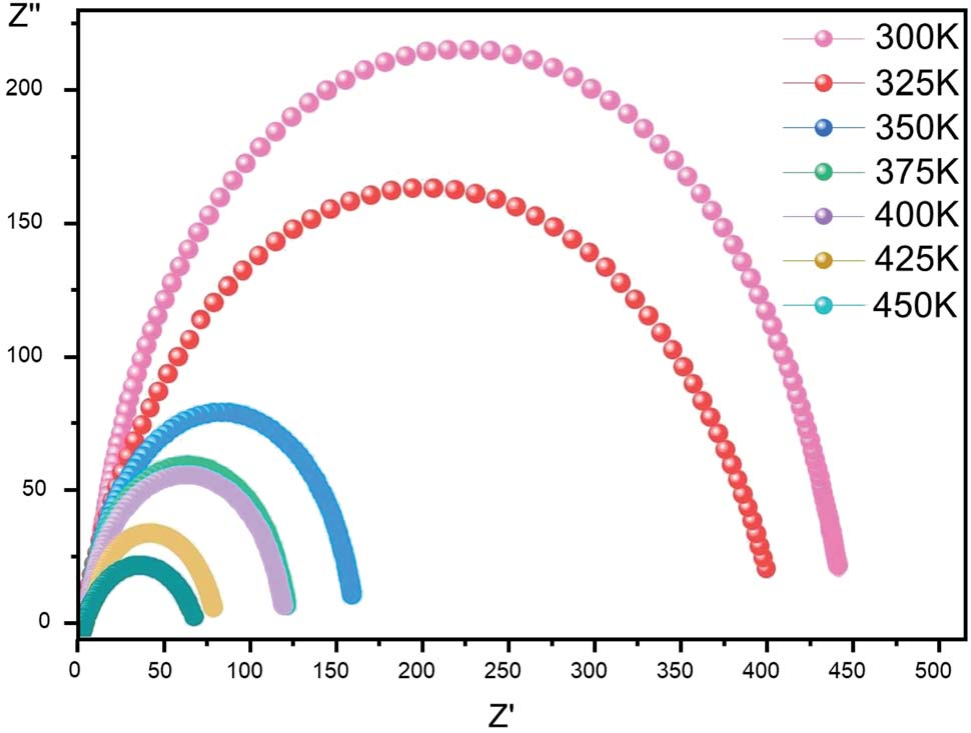
Complex impedance plot spectra of [Co^II^(TMAPP)] (2).

**Fig. 7:**
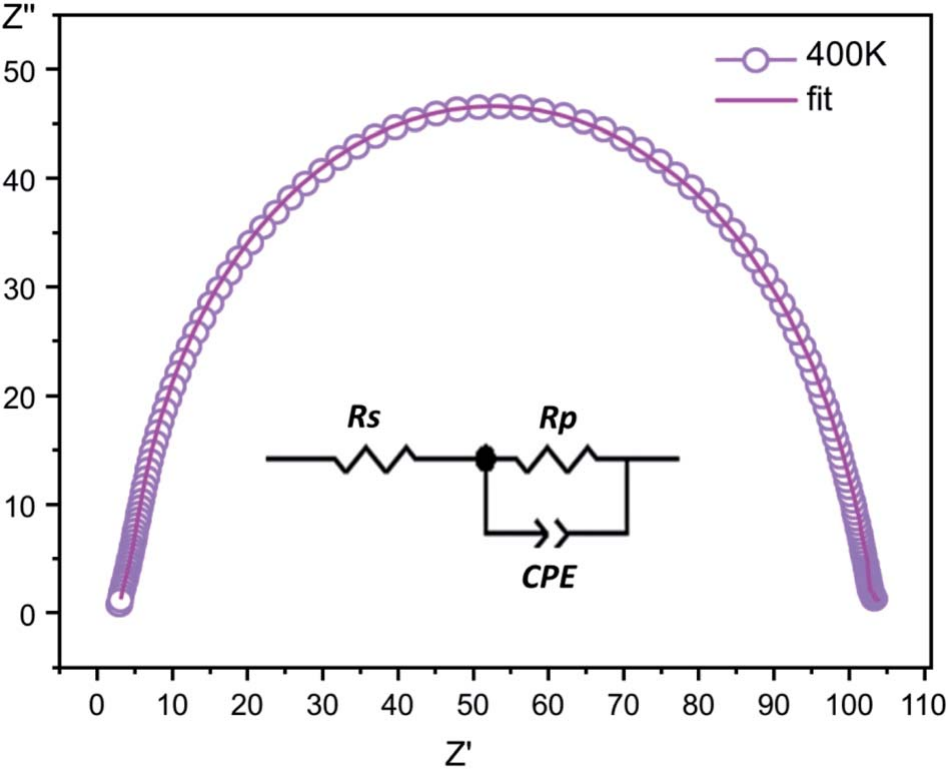
The fitted circle on the experimental data at 400 K and the equivalent circuit.

5 Z* = *Z*′ − i*Z*′′

where *Z*′ is the real part and *Z*′′ is the imaginary part. Fig. 6 shows the impedance plots of *Z*′′ with *Z*′ at various temperatures for [Co^II^(TMAPP)]. It can be observed that this spectrum at each temperature consists of a single well-shaped semicircle. Also, it can be seen that the diameters of the semicircles decrease when the temperature increases. This behaviour confirms the semiconducting characteristic of our compound in the entire explored temperature range. The radius is related to the defect states effect, which decreases with the applied voltage. These single semicircle impedance characteristics in the Cole–Cole plots can usually be reproduced with an equivalent circuit, as shown in the inset of Fig. 7. The equivalent circuit can be designed as a single parallel resistor (*R*_p_) and capacitor (CPE) network with a series resistance (*R*_s_).^52^

#### Modulus

The electrical modulus M* is expressed as:^53^

**Fig. 8:**
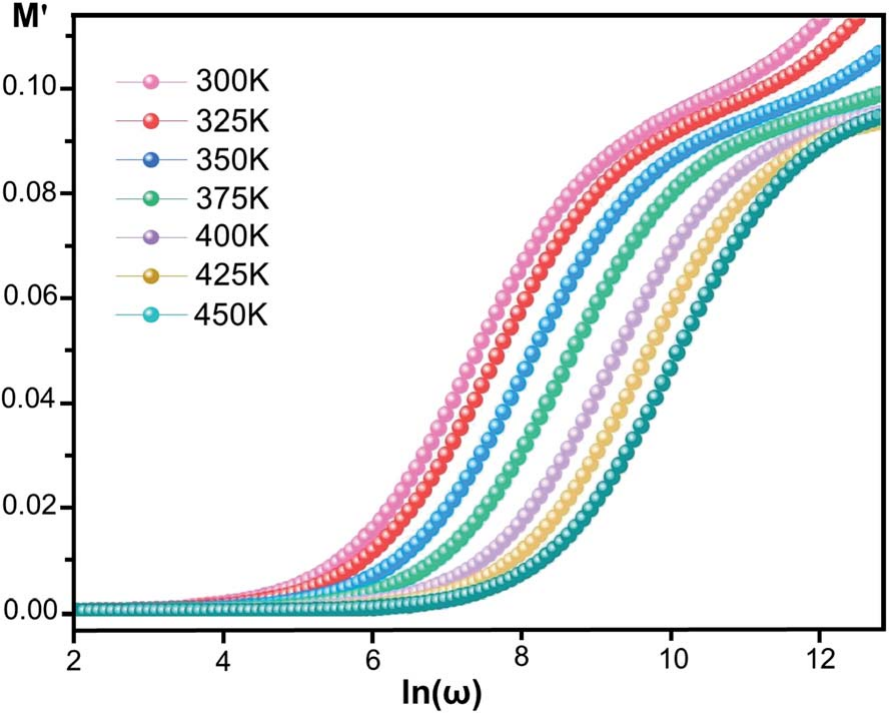
Real part of the electric modulus, *M*′, *versus* frequency at various temperatures for [Co^II^(TMAPP)].

**Fig. 9:**
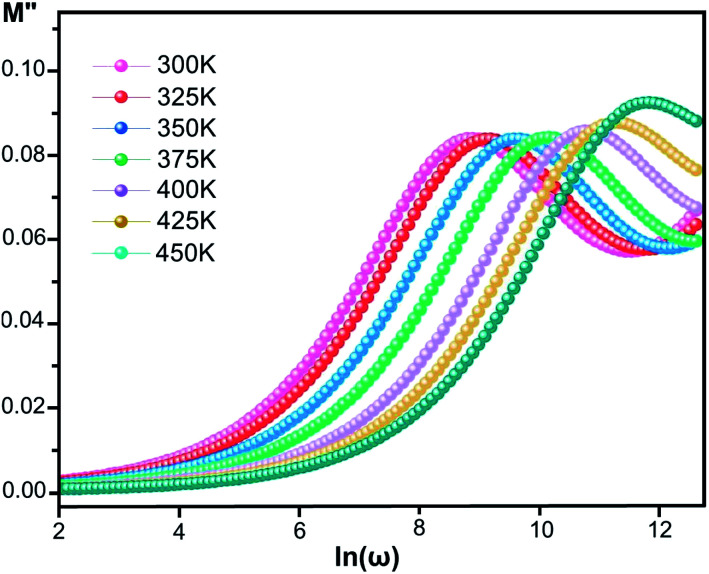
Imaginary part of the electric modulus, *M*′′, *versus* frequency at various temperatures for [Co^II^(TMAPP)].

6 M*(*ω*) = 1/ε*(*ω*) = *M*′ + i*M*′′

where ε* is the complex permittivity and *M*′ and *M*′′ are the real and the imaginary parts of the electric modulus, respectively. Fig. 8 and 9 show the frequency dependence of the real and imaginary parts of the electric modulus at various temperatures. In the lower frequency region, *M*′ has low values or shows a tendency to zero in the overall range of temperature (Fig. 8), whereas it exhibits a maximum value in the high frequency region; this can be attributed to the enhancement of the conductivity because the charge carriers are mobile through a short distance. In addition, the values of *M*′ were found to be near zero for all the given temperatures, which may be due to the suppression of electrode polarization. It can be also noted that *M*′ decreases with increasing temperature over the entire range of frequency.^54–56^ The plot of the imaginary part *M*′′ as a function of frequency (Fig. 9) shows an asymmetric maximum at all measured temperatures. The position of the peak 
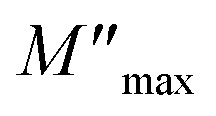
 shifts to higher frequency as the temperature increases. Two apparent distinct regions were observed and are temperature dependent. The first is located towards the left of the peak, which is associated with a conduction process where the charge carriers are mobile. The other region towards the right of the peak is related to the relaxation polarization process, where the charge carriers are spatially confined to the potential wells.

The relaxation peak 
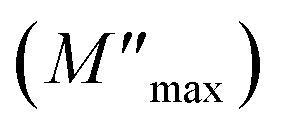
 shifts with increasing temperature, which indicates that the temperature plays an effective role in decreasing the relaxation time *τ*. The corresponding relaxation time satisfies an almost linear dependence, in accordance with the Arrhenius law:^57^

**Fig. 10:**
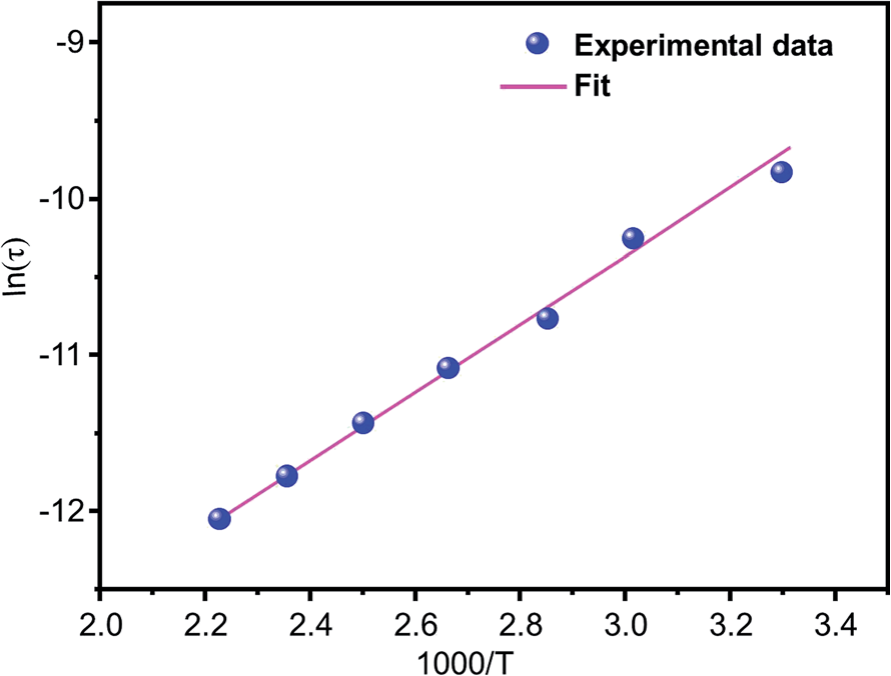
The variation of the relaxation time (*τ*) with temperature.

7 *τ* = *τ*_0_ exp(Δ*E*_*τ*_/*k*_B_*T*)

where *τ*_0_ is a constant characteristic relaxation time that represents the time of a single oscillation of a dipole in the potential well, Δ*E*_*τ*_ is the free energy of activation for dipole relaxation and *τ* (*τ* = 1/2π*f*_max_) represents the average or the most probable value of the spread of the relaxation time. The plot of ln *τ versus* 1000/*T* for the studied device is shown in Fig. 10. The value of the activation energy Δ*E*_*τ*_ was calculated from the slope and the intercept of the linear fit and was found to be 0.319 eV.

The activation energies deduced from the conductivity and electrical modulus curves are close, demonstrating that the mechanism of relaxation is the same and that the origin of relaxation may be related to the thermally activated behavior of the same charge carriers.

### 3.6. Adsorption studies

#### Selective adsorption of cationic dyes

**Fig. 11:**
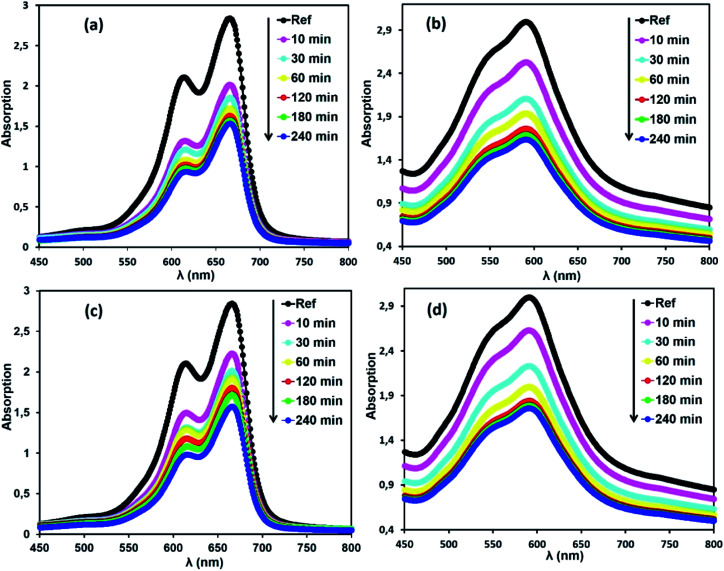
Changes in the UV-vis absorption spectra of MB and CV after addition of the adsorbents, H_2_TMAPP (*m* = 3 mg) [(a) and (b), respectively] and [Co^II^(TMAPP)] (*m* = 3 mg) [(c) and (d), respectively]. The concentrations of MB and CV are *C*_0_ = 30 mg L^−1^, pH = 6 and *T* = 298 K.

To evaluate the properties of the free porphyrin and metalloporphyrin as adsorbents, adsorption testing was conducted with methylene blue (MB) and crystal violet (CV) as model dyes. To examine the adsorption abilities of 1 and 2 for these dyes, samples of 1 and 2 (*m* = 3 mg) were dipped into an aqueous solution of MB and CV (*C*_0_ = 30 mg L^−1^) at room temperature (*T* = 298 K), and the adsorption process was detected by UV-vis spectra (Fig. 11). After compounds 1 and 2 adsorbed the dyes, the UV spectra of the aqueous solutions showed that the characteristic peaks for MB and CV at 664 and 591 nm, respectively, decreased gradually for both compounds, indicating the removal of these dyes.

**Fig. 12:**
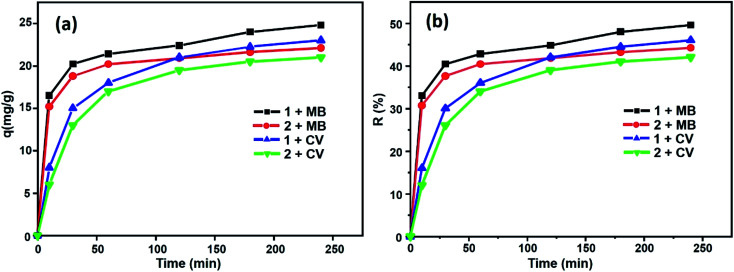
Evolution of (a) the adsorption capacities for MB and CV and (b) yield removal *versus* time.

Fig. 12 shows the percentages of adsorption of MB and CV dyes on compounds 1 and 2 using MB dye as the adsorbate (Fig. 12a, black and red). For the free porphyrin, the adsorption rate was found to increase rapidly within the first 15 min and remained high up to 240 min. Similarly, for compound 2, the adsorption rate was found to increase rapidly; however, it always remained inferior to that of the free porphyrin. In the case of CV dye as the adsorbate (Fig. 12a, blue and green), the trend of the adsorption curve was different compared to that of MB. The rate of the CV adsorption was found to be lower than MB within the first 15 min and continued to increase during the course of the reaction. The adsorption capacity was determined using eqn (8):^58^

8 *q*_*t*_ = (*C*_0_ − *C*_*t*_)*V*/*m*

Here, *C*_0_ (mg L^−1^) and *C*_*t*_ (mg L^−1^) are the liquid-phase concentrations of the dye at the beginning and after equilibrium time *t* [min], respectively; *V* (L) is the volume of the solution; and *m* [g] is the mass of the materials used. The percentage of dye removal from aqueous solution was determined according to eqn (9):^59^9
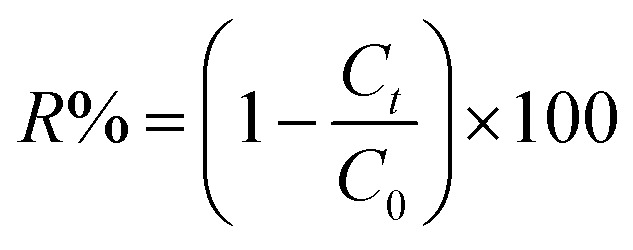
where *C*_0_ = the initial concentration of dye (mg L^−1^) and *C*_*t*_ = the dye concentration (mg L^−1^) at time *t*.

From this equation and under the experimental conditions of room temperature, *C*_0_ = 30 mg L^−1^, *m* = 3 mg and pH 6, we found that about 42% and 44.2% of the studied dyes were removed from CV and MB, respectively, for the Co-porphyrin (2), while for the free porphyrin (1), the returns were 49.6% and 46% for MB and CV, respectively (Fig. 12b). Our porphyrin is rich in oxygen and nitrogen atoms, which aided the adsorption. Thus, intermolecular interactions occurred between the dye molecules and functional groups (oxygen and nitrogen) of the porphyrin. It is reasonable to assume that these intermolecular hydrogen bonding interactions are responsible for the selective dye adsorption, thus effectively removing MB molecules. The number of functional groups (nitrogen functions) decreased from the free to the metallated porphyrin, that is to say, when inserting cobalt into the porphyrin core.

#### Adsorption kinetics

**Fig. 13:**
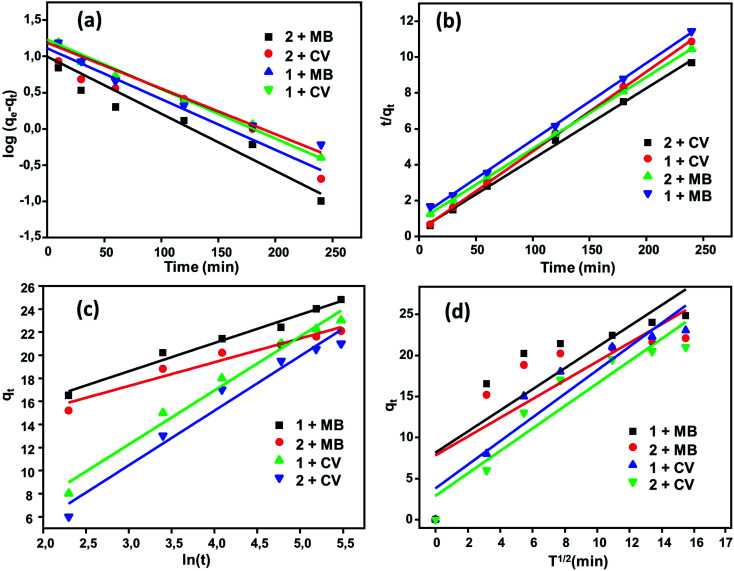
Kinetic data fitted to the (a) first order and (b) pseudo-second-order equations with fitting to the models of (c) Elovich and (d) intra-particular diffusion.

The kinetics of the adsorption process is one of the most important parameters to study to evaluate the adsorption efficiency of adsorbent materials. A comparative study of the kinetics of adsorption of the two dyes MB and CV upon H_2_TMAPP and [Co^II^(TMAPP)] was performed by applying four models, including the pseudo-first-order model, pseudo-second-order model, intra-particle diffusion model, and Elovich model. The results of the fits of the experimental data are shown in Fig. 13 and Table 1.

**Table 1:** Kinetic data for the adsorption of MB and CV dyes using compounds 1 and 2 (*C*_0_ = 30 mg L^−1^, pH = 6, *m* = 3 mg)^a^

Kinetic equations	Calculated parameters	MB		CV	
		1	2	1	2
	*q*_exp_ (mg g^−1^)	25	22.2	23.4	21.6
Pseudo first order	*k*_1_ (min^−1^)	0.016	0.018	0.016	0.014
	*q* (mg g^−1^)	12.88	9.915	17.06	15.29
	*R* ^2^	0.919	0.917	0.982	0.964
Pseudo second order	*k*_2_ (g mg^−1^ min^−1^)	0.0043	0.0069	0.0018	0.0017
	*q*_cal_ (mg g^−1^)	25.31	22.47	25	23.31
	*R* ^2^	0.998	0.999	0.999	0.999
Elovich	*α* (mg g^−1^ min^−1^)	230.55	459.77	3.17	2.14
	*β* (g mg^−1^)	0.406	0.486	0.214	0.211
	*R* ^2^	0.980	0.954	0.981	0.967
Intra-particular diffusion	*k*_d_ (mg g^−1^ min^−1/2^)	1.28	1.14	1.43	1.36
	*R* ^2^	0.699	0.662	0.891	0.893
	*C* (mg g^−1^)	8.16	7.82	3.78	2.89

a *k*_1_ = the pseudo first order rate constant (min^−1^), *k*_2_ = the second pseudo order rate constant (g mg^−1^ min^−1^), *α* (mg g^−1^ min^−1^) = the initial adsorption rate, *β* (g mg^−1^) = the desorption constant related to the extent of surface coverage and activation energy for chemisorption, *k*_d_ = the intra-particle diffusion rate constant (mg g^−1^ min^−1/2^).

The pseudo-first-order and pseudo-second-order kinetic models for the dyes (MB and CV) adsorbed over (1) and (2) in aqueous phases can be expressed as follows (Fig. 13a and b). By applying the pseudo-first-order model, the *q*_e_ values calculated are smaller than the experimental *q*_e_, and the values of the correlation coefficient [*R*^2^ = 0.919 (H_2_TMAPP), 0.917 [Co^II^(TMAPP)] for MB and *R*^2^ = 0.982 (H_2_TMAPP), 0.964 [Co^II^(TMAPP)] for CV] suggest that the model does not fit the adsorption process for the present porphyrin. For the pseudo-second-order model, as shown in Fig. 13b, the plots of *t*/*q*_*t*_ against *t* for the adsorption kinetics of MB and CV over the adsorbents H_2_TMAPP and [Co^II^(TMAPP)] gave straight lines that were well-fitted to the experimental data with high correlation coefficients [*R*^2^ = 0.998 (H_2_TMAPP), 0.999 [Co^II^(TMAPP)] for MB and *R*^2^ = 0.999 (H_2_TMAPP), 0.999 [Co^II^(TMAPP)] for CV], confirming a high degree of accuracy for the adsorptions. These results imply that the adsorption kinetics can be best described by a pseudo-second-order model for both H_2_TMAPP and [Co^II^(TMAPP)].

The diffusion-based model, intra-particle diffusion model and Elovich model were then fitted to determine the diffusion mechanisms of MB and CV adsorption on (1) and (2) (Fig. 13c and d). According to Table 1, the correlation coefficients for intraparticle diffusion [*R*^2^ = 0.699 (H_2_TMAPP), 0.662 [Co^II^(TMAPP)] for MB and *R*^2^ = 0.891 (H_2_TMAPP), 0.893 [Co^II^(TMAPP)] for CV] were lower than those for pseudo-second-order kinetics. This again suggests that the pseudo-second-order adsorption mechanism is predominant and that the overall rate of the dye adsorption process appears to be controlled by more than one step.

### 3.7. The catalytic degradation of methylene blue and crystal violet

**Fig. 14:**
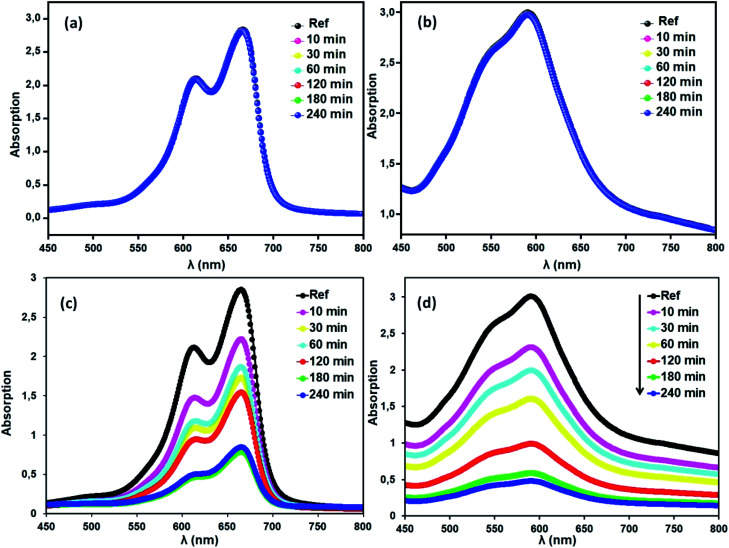
Blank tests: (a) (MB), (b) (CV). Evolution of the absorbance *versus* time using the compound (2) (3 mg)/H_2_O_2_ (6 mg L^−1^) system (c) with MB and (d) with CV. The concentrations of MB and CV were 30 mg L^−1^, pH = 6 and *T* = 298 K.

**Fig. 15:**
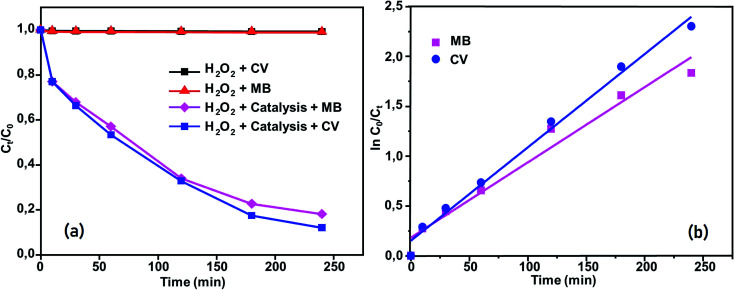
(a) Changes in *C*_*t*_/*C*_0_*versus* time for the following conditions: dyes + H_2_O_2_, MB + compound (2) + H_2_O_2_ and CV + compound (2) + H_2_O_2_. (b) Kinetics of [Co^II^(TMAPP)]-catalyzed degradation of MB and CV in aqueous solution.

Methylene blue (MB) and crystal violet (CV), two typical industrial pollutants, were chosen as model dyes to examine the catalytic behaviors of the as-synthesized Co-porphyrin. The oxidative degradation of the dyes was investigated under neutral conditions in the presence of H_2_O_2_ and [Co^II^(TMAPP)] as the catalyst. Fig. 14 shows the advancement of catalytic degradation of the dyes monitored by the reduction in absorbance at the respective *λ*_max_ values of the dyes at different times (10, 30, 60, 120, 180 and 240 min). Fig. 15a depicts the changes in the *C*_*t*_/*C*_0_ values of MB and CV (*C*_0_ = 30 mg L^−1^) using different combinations. Before the degradation process, MB showed two absorption peaks centered at 664 nm (Fig. 14a). Similarly, CV exhibited two peaks at 591 nm (Fig. 14b). After the addition of H_2_O_2_ (6 mg L^−1^) alone without the addition of any product, the dye solutions were stable. As [Co^II^(TMAPP)] was added, the intensities of the peaks decreased rapidly for MB at 664 nm and for CV at 591 nm.

Moreover, the addition of this compound to the dye solutions leads to a decrease in the concentration. Using compound 2, only 42% and 44.2% dye discoloration was achieved for CV and MB, respectively. However, using the compound 2/H_2_O_2_ system (dye concentration = 30 mg L^−1^, pH 6 and *t* = 4 hours of interaction), the value of the discolouration yield increased rapidly, reaching 90% for CV and 84% for MB at equilibrium. This difference in decolourisation can be explained by the fact that MB dye degrades more easily than CV. The presence of hydrogen peroxide accelerates the elimination of the dye in the solution. To this end, we proposed a catalytic mechanism based on three stages (adsorption–oxidation–desorption). The adsorption phase consists of the fixation of dyes and hydrogen peroxide on the surface of the catalyst. Hydrogen peroxide in contact with complex 2 released many more reactive hydroxyl radicals, which degrade BM and CV. Above all, it was observed that the MB and CV solutions seemed to be stable in the presence of H_2_O_2_ alone. These behaviors were detected when investigating the degradation of textile dyes using chitosan-supported [bis(2-methylallyl)(1,5-cyclooctadiene)ruthenium(ii)].^60^ According to the literature, the degradation yield of BM dye obtained using our Co(ii)-porphyrin compound (84%) is comparable and in some cases even superior to those of previously synthesized and studied cobalt metalloporphyrins.^17^ On the other hand, this degradation yield is lower than those obtained with metalloporphyrin polymers (99%).^61^ For CV dye degradation, the yield obtained (90%) is quite acceptable for the first reported investigation of the degradation of this dye using a porphyrin derivative.

The reactions of BM and CV solutions at 30 mg L^−1^ with hydrogen peroxide have high correlations of *R*^2^ = 0.98 and *R*^2^ = 0.96, respectively. This confirms that the reaction follows the pseudo-second-order kinetic model. To provide insight into the dye degradation kinetics, the constant *k*_0_ was computed:10
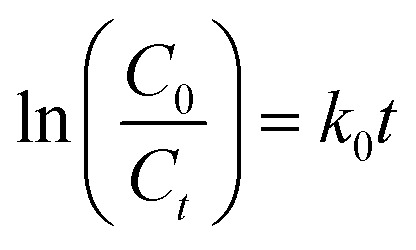
where *t* is the time taken during the degradation, *k*_0_ is the first-order rate constant of the reaction, and *C*_*t*_ and *C*_0_ are the dye levels at times *t* and 0. As depicted in Fig. 15b, the rate constants of the degradation (*k*_0_)^62^ are 0.009 and 0.007 min^−1^ for CV and MB, respectively.

The catalyst was regenerated efficiently at the end of the catalytic process and remained stable, which was confirmed by IR (Fig. S7†), and no change was observed.

## 4. Conclusions

In summary, the synthesis and determination of the spectral and optical properties of the new porphyrin H_2_TMAPP (1) and its cobalt complex [Co^II^(TMAPP)] (2) were performed. The UV-visible spectrum of 2 exhibits a blue-shifted Soret band compared to the free porphyrin, and the fluorescence data are practically the same as those of known cobalt porphyrin complexes. The position and sharpness of the ^1^H NMR resonances clearly demonstrate the paramagnetic nature of the complex, indicative of a Co(ii) complex. The complex impedance was investigated by an equivalent circuit constituted by a resistor (*R*_p_) in parallel with a capacitor (CPE), all in series with a resistor (*R*_s_). The dielectric modulus confirmed the presence of a non-Debye relaxation type. The activation energies, determined from the conductivity and the electrical modulus, are comparable, which proves that the relaxation phenomenon is the same and that the origin of relaxation may be related to the thermally activated behavior of the same charge carriers. The present compounds show good adsorption properties toward two dyes (methylene blue and crystal violet) that are present in organic pollutants. The kinetic data disclosed the pseudo-second order mechanism of adsorption. Interestingly, the aggregate of [Co^II^(TMAPP)] (2) exhibits higher catalytic efficiency for the degradation of cationic dyes in the presence of H_2_O_2_.

## Conflicts of interest

There are no conflicts of interest to declare.

## Supplementary Material

RA-010-D0RA08786F-s001

## References

[cit1] Valicsek Z., Horváth O., Patonaya K. (2011). J. Photochem. Photobiol., A.

[cit2] Ahmad S., Gautam R., Singhal A., Chauhan S. M. S. (2018). J. Mol. Liq..

[cit3] Mishra J., Pattanayak D. S., Das A. A., Mishra D. K., Rath D., Sahoo N. K. (2019). J. Mol. Liq..

[cit4] Crossley M. J., Burn P. L. (1991). J. Chem. Soc., Chem. Commun..

[cit5] Kou J., Dou D., Yang L. (2017). Oncotarget.

[cit6] Sugiyasu K., Takeuchi M. (2009). Chem.–Eur. J..

[cit7] Song H., Liu Q., Xie Y. (2018). Chem. Commun..

[cit8] Ray A., De A., Bhattacharya S. (2017). J. Mol. Liq..

[cit9] Shaikh S. M., Chakraborty A., Alatis J., Caia M., Danilov E., Morris A. J. (2019). Faraday Discuss..

[cit10] Leishman C. W., McHale J. L. (2015). J. Phys. Chem. C.

[cit11] Otsuki J. (2018). J. Mater. Chem. A.

[cit12] Oviedo O., Zoltan T., Vargas F., Marcel I., Vivas J. C. (2014). J. Coord. Chem..

[cit13] Carminati D. M., Intrieri D., Le Gac S., Roisnel T., Boitrel B., Toma L., Legnanic L., Galloa E. (2017). New J. Chem..

[cit14] Isaac M. F., Kahl S. B. (2003). J. Organomet. Chem..

[cit15] Mohajer D., Karimipour G., Bagherzadeh M. (2004). New J. Chem..

[cit16] Karimipour G., Karami B., Montazerozohori M., Zakavi S. (2007). Chin. J. Catal..

[cit17] Guergueb M., Nasri S., Brahmi J., Loiseau F., Molton F., Roisnel T., Guerineau V., Turowska-Tyrk I., Aouadi K., Nasri H. (2020). RSC Adv..

[cit18] Amiri N., Nouir S., Hajji M., Roisnel T., Guerfel T., Simonneaux G., Nasri H. (2019). J. Saudi Chem. Soc..

[cit19] Amiri N., Hajji M., Ben Taheur F., Chevreux S., Roisnel T., Lemercier G., Nasri H. (2018). J. Solid State Chem..

[cit20] Soury R., Jabli M., Saleh T. A., Abdul-Hassan W. S., Saint-Aman E., Loiseau F., Philouze C., Nasri H. (2018). RSC Adv..

[cit21] Perrin D. D. and Armarego W. L. F., Purification of Organic Solvents, Pergamon Press, Oxford, 1988

[cit22] Sharma V. S., Sharma A. S., Vekariya R. H., Patel R. B. (2017). Mol. Cryst. Liq. Cryst..

[cit23] Lindsey J. S., Hsu H. C., Schreiman I. C. (1986). Tetrahedron Lett..

[cit24] Adler A. D., Longo F. R., Kampas F., Kim J. (1970). J. Inorg. Nucl. Chem..

[cit25] Pessoa C. A., Gushikem Y. (2001). J. Porphyrins Phthalocyanines.

[cit26] Forgues S., Lavabre D. (1999). J. Chem. Educ..

[cit27] Maree M. D., Nyokong T., Suhling K., Phillips D. (2002). J. Porphyrins Phthalocyanines.

[cit28] Ezzayani K., Ben Khelifa A., Saint-Aman E., Loiseau F., Nasri H. (2017). J. Mol. Struct..

[cit29] Farran R., Jouvenot D., Loiseau F., Chauvin J., Deronzier A. (2014). Dalton Trans..

[cit30] Mansour A., Belghith Y., Belkhiria M. S., Bujaczb A., Guérineau V., Nasri H. (2013). J. Porphyrins Phthalocyanines.

[cit31] Shaffer A. M., Gouterman M. (1972). Theor. Chim. Acta.

[cit32] Gouterman M. (1959). J. Chem. Phys..

[cit33] Lyons D. M., Kesters J., Maes W., Christopher W., Bielawski J., Sessler L. (2013). Synth. Met..

[cit34] Ezzayani K., Ben Khelifa A., Saint-Aman E., Loiseau F., Nasri H. (2016). Polyhedron.

[cit35] Yan B., Liu X., Ghugare T., Fedorka N., Li Y. F. (2015). J. Coord. Chem..

[cit36] Chen W., Fukuzumi S. (2009). Eur. J. Inorg. Chem..

[cit37] Du Y. X., Zhang Z. Q., Yao Y. H., Li J. (2016). Inorg. Chem. Commun..

[cit38] Patwari J., Chatterjee A., Sardar S., Lemmens P., Pal S. K. (2018). Phys. Chem. Chem. Phys..

[cit39] Kadish K. M., Morrison M. M. (1976). J. Am. Chem. Soc..

[cit40] Ke X., Kumar R., Sankar M., Kadish K. M. (2018). Inorg. Chem..

[cit41] Kadish K. M., Mu X. H., Lin X. Q. (1988). Inorg. Chem..

[cit42] Truxillo L. A., Davis D. G. (1975). Anal. Chem..

[cit43] Paul-Roth C., Rault-Berthelot J., Simonneaux G., Poriel C., Abdalilah M., Letessier J. (2006). J. Electroanal. Chem..

[cit44] El-Nahassa M. M., Metwallya H. S., El-Sayeda H. E. A., Hassaniena A. M. (2012). Mater. Chem. Phys..

[cit45] Badri A., Jabli M., López M. L., Ben Amara M. (2019). Inorg. Chem. Commun..

[cit46] Masoud M. S., Ali A. E., Mostafa M. A. E.-Z., Mohamed R. H. (2005). Spectrochim. Acta, Part A.

[cit47] Kakade S. G., Ma Y.-R., Devan R. S., Kolekar Y. D., Ramana C. V. (2016). J. Phys. Chem. C.

[cit48] Jonscher A. K. (1977). Nature.

[cit49] Elliott S. R. (1987). Adv. Phys..

[cit50] Shehata M. M., Abdel-Malik T. G., Abdelhady K. (2018). J. Alloys Compd..

[cit51] El-Nahass M. M., Zeyada H. M., Makhlouf M. M. (2011). Appl. Phys..

[cit52] Ceyhan T., Altındal A., Erbil M. K., Bekaroglu Ö. (2006). Polyhedron.

[cit53] Ranjan R., Kumar R., Kumar N., Behera B., Choudhary R. N. P. (2011). J. Alloys Compd..

[cit54] Afandiyeva I. M., Dökme I., Altındal S., Bülbül M. M., Tataro glu A. (2008). Microelectron. Eng..

[cit55] Aziz S. B., Abidin Z. H. Z. (2015). J. Appl. Polym. Sci..

[cit56] Elliott S. R. (1988). Solid State Ionics.

[cit57] El-Nahass M. M., Farag A. A. M., Abu-Samaha F. S. H., Elesh E. (2014). Vacuum.

[cit58] Lv L.-L., Yang J., Zhang H.-M., Liu Y.-Y., Ma J.-F. (2015). Inorg. Chem..

[cit59] Khan M. S., Khalid M., Ahmad M. S., Shahid M., Ahmad M. (2019). Dalton Trans..

[cit60] Jabli M., Touati R., Kacem Y., Hassine B. B. (2012). J. Text. Inst..

[cit61] Li Y., Wang L., Gao Y., Yang W., Li Y., Guo C. (2018). RSC Adv..

[cit62] Sharma V. S., Sharma A. S., Vekariya R. H., Patel R. B. (2017). Mol. Cryst. Liq. Cryst..

