# Supplementary material for: A new cobalt(ii) meso-porphyrin: synthesis, characterization, electric properties and application in the catalytic degradation of dyes
Source: RSC Adv. 2020 Dec 18;10(73):44920–32. doi: 10.1039/d0ra08786f (PMC9058613; doi:10.1039/d0ra08786f)
Supplement: RA-010-D0RA08786F-s001 [file RA-010-D0RA08786F-s001.pdf]

Electronic Supplementary Material (ESI) for RSC Advances.
This journal is © The Royal Society of Chemistry 2020

Electronic Supplementary Information (ESI)

A new cobalt(II) meso-porphyrin: Synthesis, characterization, electric property and application in catalytic degradation of dyes

Nesrine Amiri[a, *], Mouhieddinne Guergueb[a], Maged S. Al-Fakeh[b], Marwa Bourguiba[c,d] and Habib Nasri[a]

[a] *Laboratory of Physical Chemistry of Materials, University of Monastir, Avenue of the Environment, 5019 Monastir, Tunisia.*
[b] *Department of Chemistry, Faculty of Science, Qassim University, Saudi Arabia.*
[c] *Applied Mechanics and Systems Research Laboratory (LASMAP-EPT), polytechnic school, La Marsa, University of carthage, Tunisia.*
[d] *Faculty of science of Tunis, University of Tunis el manar, Tunisia.*

Table of contents

No.	Contents	Pg No.
1	Scheme S1: Main chemical preparation steps.	2
2	Table S1: UV/Vis data of several free base *meso*-porphyrins, H_2TMAPP (**1**), Co^{II}TMAPP (**2**) and a selection of cobalt *meso*-metalloporphyrins in dichloromethane.	3
3	Table S2: Electrochemical data for H_2TMAPP (**1**), [Co^{II}(TMAPP)] (**2**) and a selection of *meso*-porphyrins and Co(II) *meso*-metalloporphyrins. All data are obtained from voltammograms recorded in dichloromethane.	4
4	Fig. S1: IR spectrum of H_2TMAPP (**1**).	5
5	Fig. S2: IR spectrum of Co^{II}TMAPP (**2**).	5
6	Fig. S3. ^{1}HNMR spectrum of H_2TMAPP (**1**).	6
7	Fig. S4. ^{1}HNMR spectrum of Co^{II}TMAPP (**2**).	6
8	Fig. S5. Positive MALDI-TOF mass spectrum of H_2TMAPP (**1**).	7
9	Fig. S6. Positive MALDI-TOF mass spectrum of [Co^{II}(TMAPP)] (**2**).	7
10	Fig. S7. FT-IR spectra of [Co^{II}(TMAPP)]: (a) before degradation, (b) after degradation of MB, and (c) after degradation of CV.	8
11	References	9

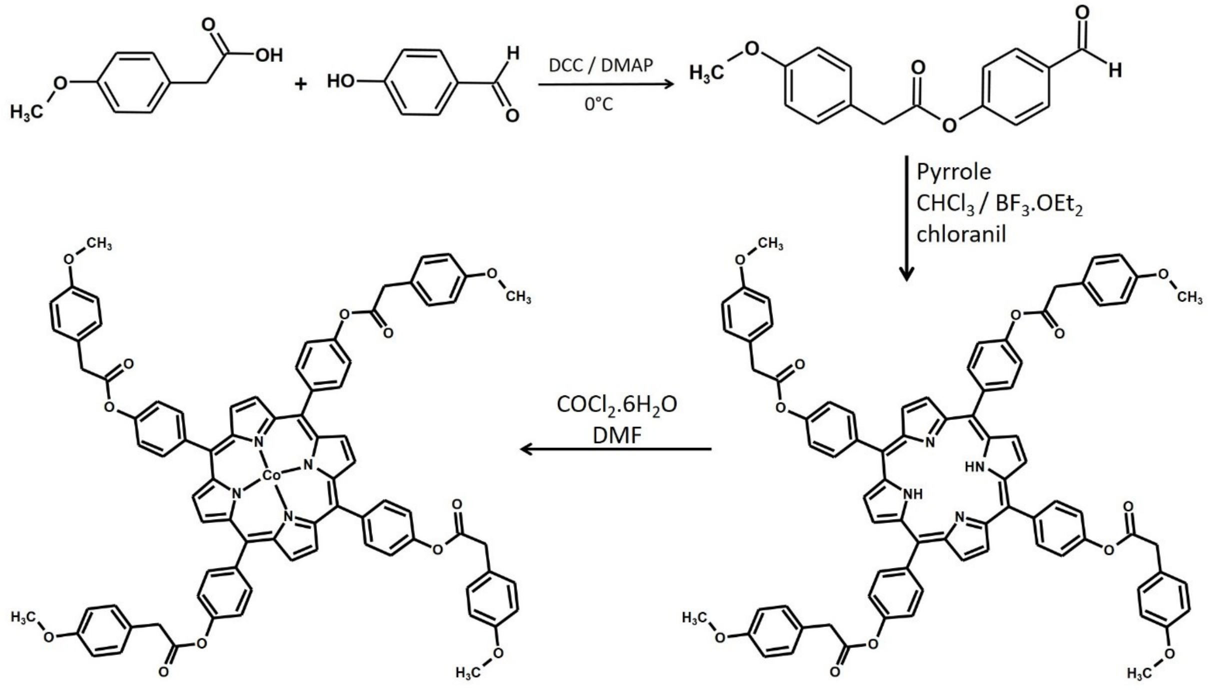

Scheme S1. Main chemical preparation steps.

Table S1: UV/Vis data of several free base meso-porphyrins, H_2TMAPP (1), Co^{II}TMAPP (**2**) and a selection of cobalt *meso*-metalloporphyrins in dichloromethane.

**Compound**	***Soret* band** **λ [nm] (log ε)**	**Q bands** **λ [nm] (log ε)**				**[ref]**
		Meso-porphyrins				
**H_2TPP**	416 (6.10)	513 (5.70)	550 (4.36)	590 (4.24)	646 (4.19)	[1]
**H_2TTP**	420 (5.95)	515 (5.82)	555 (4.28)	595 (4.17)	650 (4.10)	[1]
**H_2TBrPP**	419 (6.58)	515 (5.24)	549 (4.93)	590 (4.77)	648 (4.68)	[2]
**H_2TPBP**	419 (5.90)	515 (4.46)	551 (4.13)	590 (3.94)	646 (3.84)	[3]
**H_2Pp**	421	517	553	592	648	[4]
**H_2TMAPP**	425 (5.92)	522(5.67)	550 (4.32)	597 (4.15)	653 (3.98)	this work
		Co(II)-meso-porphyrins				
**[Co^{II}(TPP)]**	412	528				[5]
**[Co^{II}(TpivPP)]**	412	524				[6]
**[Co^{II}(TPBP)]**	412 (6.07)	528 (5.58)				[7]
**[Co(II)(Pp)]**	414	531				[4]
**[Co^{II}(TMAPP)]**	415 (5.85)	539 (5.49)				this work

Table S2. Electrochemical data [a] for H_2TMAPP, [Co^{II}(TMAPP)] and a selection of *meso*-porphyrins and Co(II) *meso*-metalloporphyrins. All data are obtained from voltammograms recorded in dichloromethane.

	Oxidations				Reductions			
	1st Porph oxid. (O1,R1)	2nd Porph oxid. (O2,R2)	3rd Porph oxid. (O3,R3)	Oxid.Co(II)/ Co(III) (MO2)	1st Porph red. (R4,O4)	2nd Porph red. (R5,O5)	Red Co(II)/ Co(I) (MR1[b],MO1[c])	
	$E_{1/2}$[d]	$E_{1/2}$	$E_{1/2}$	$E_{1/2}$	$E_{1/2}$	$E_{1/2}$	$E_{1/2}$	Ref.
**H_2TPP**	1.02	1.26	—	—	-1.20	-1.55	—	[8]
**H_2TMPP**	1.02	1.19	1.67[c]	—	-1.19	-1.52	—	[9]
**H_2TClPP**	1.00	1.23	1.53	—	-1.09	-1.41	—	[9]
**H_2TMAPP**	1.15	1.46	1.66	—	-1.08	-1.41	—	This work
**[Co^{II}(TPP)]**	1.16	—	—	0.98	-1.40[e]	—	-0.83	[10]
**[Co^{II}(TPP)]**	0.97	—	—	0.78	—	—	-0.85	[11]
**[Co^{II}(TPP)]**	0.91	—	—	0.75	—	—	—	[12]
**[Co^{II}(TClPP)]**	1.00	1.26	1.85	0.60[c]	-1.40	—	-0.88	[9]
**[Co^{II}(TMPP)]**	0.93	1.20	—	0.70[c]	-1.36	—	-0.70	[9]
**[Co^{II}(TMAPP)]**	1.08	1.29	—	0.61	-1.38	—	-0.93	This work

[a] The potentials are reported versus SCE. [b] MR Metal Reduction. [c] MO Metal Oxidation. [d] $E_{1/2}$ half wave potential. [e] Irreversible wave.

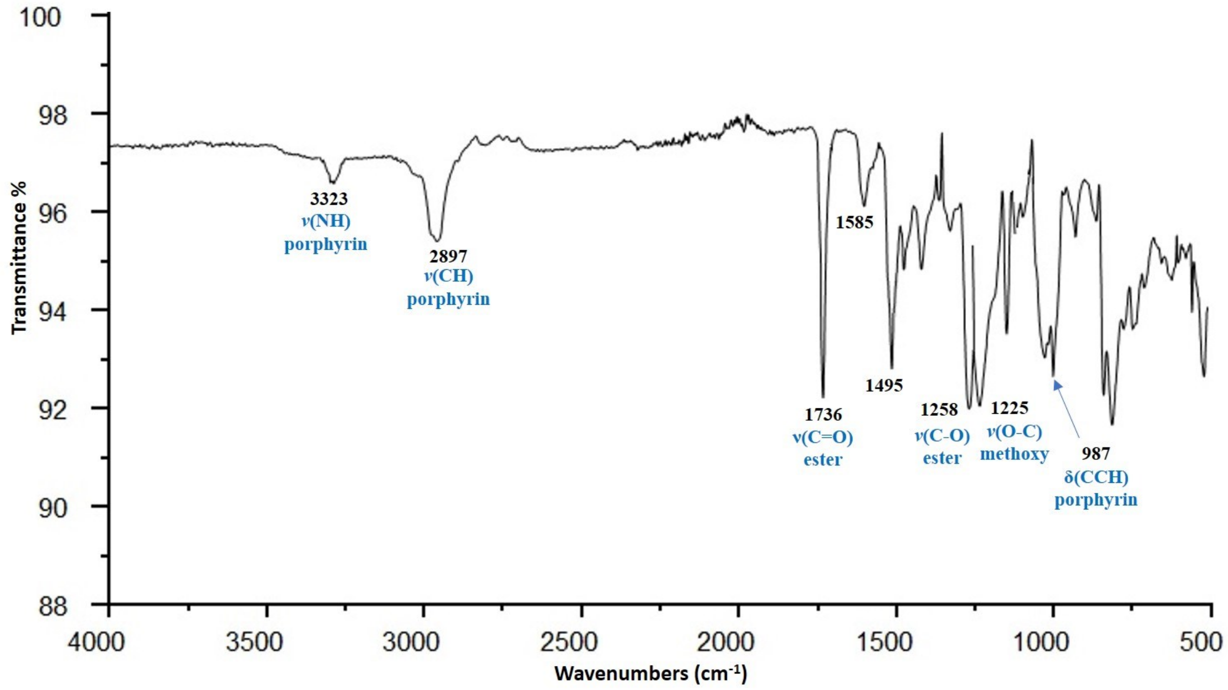

Fig. S1. IR spectrum of (**1**).

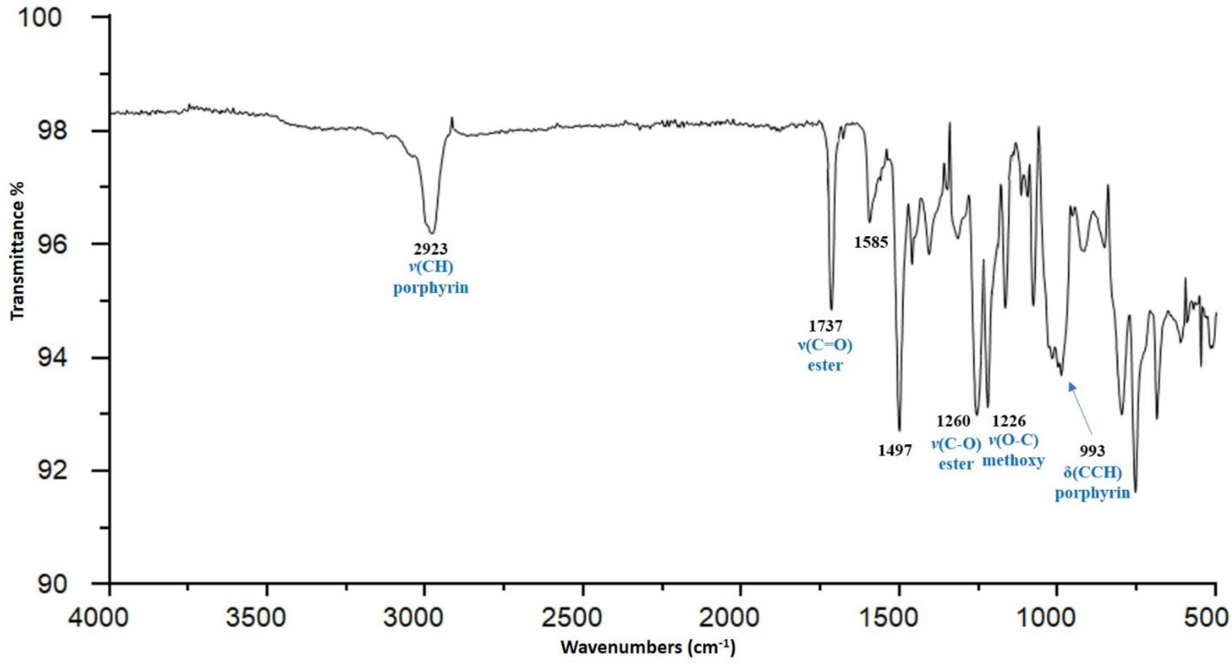

Fig. S2. IR spectrum of (**2**).

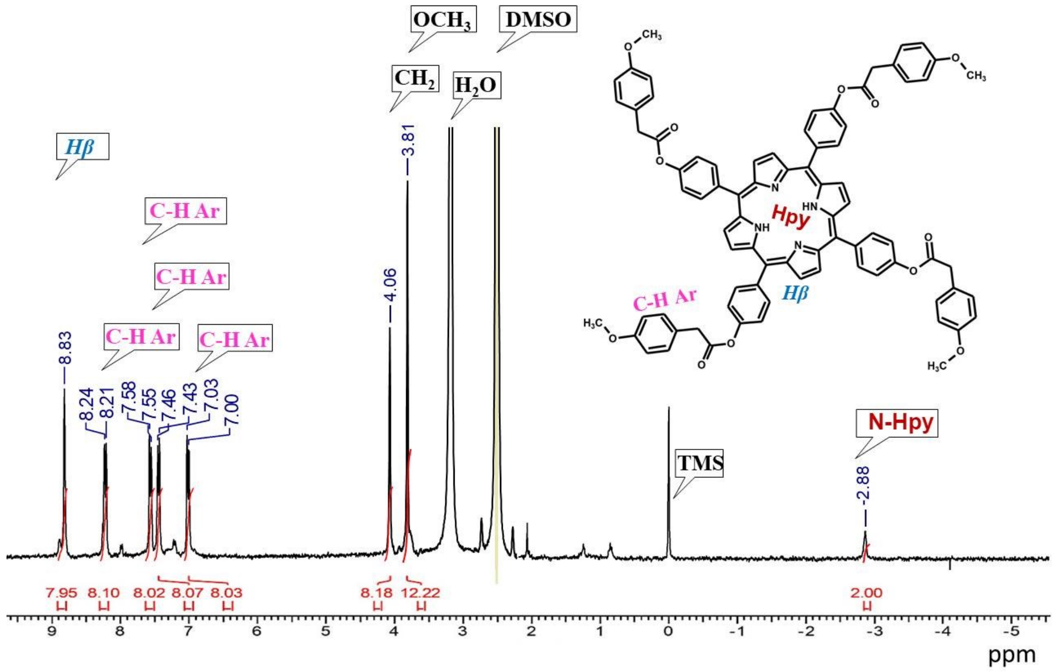

Fig. S3. ^{1}HNMR spectrum of H_2TMAPP (**1**).

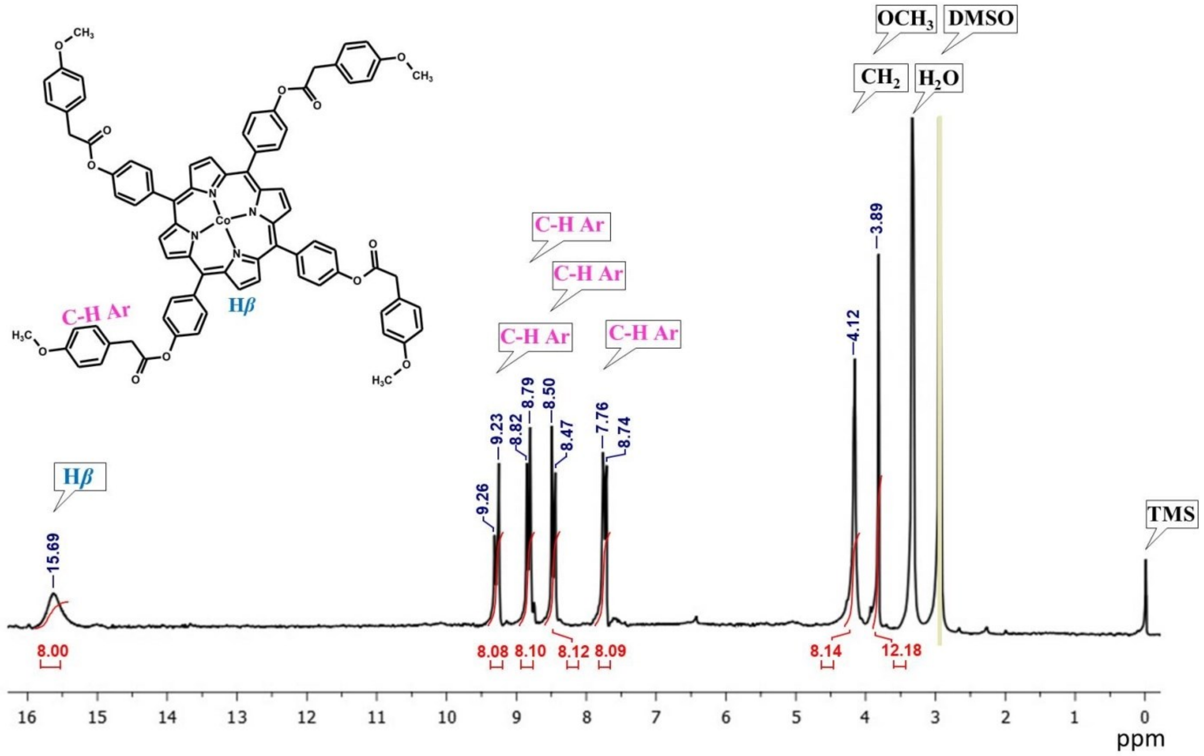

Fig. S4. ^{1}HNMR spectrum of Co^{II}TMAPP (**2**).

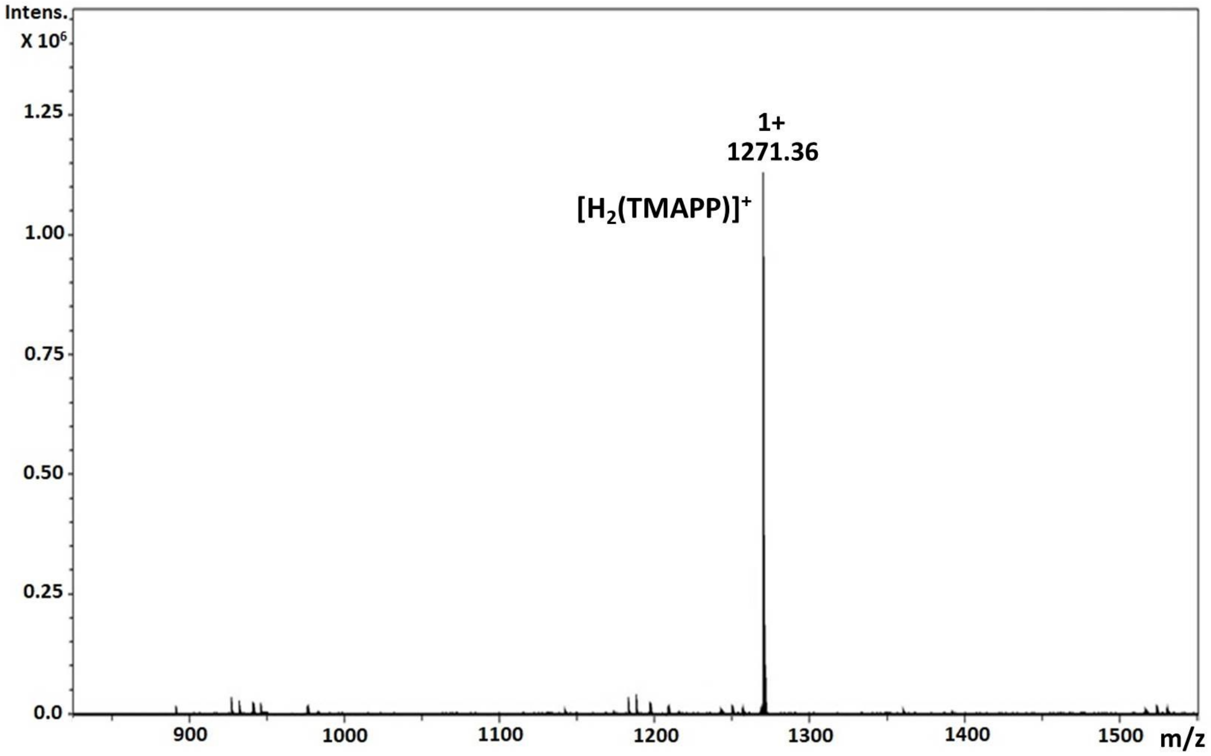

Fig. S5. Positive MALDI-TOF mass spectrum of H_2TMAPP (**1**).

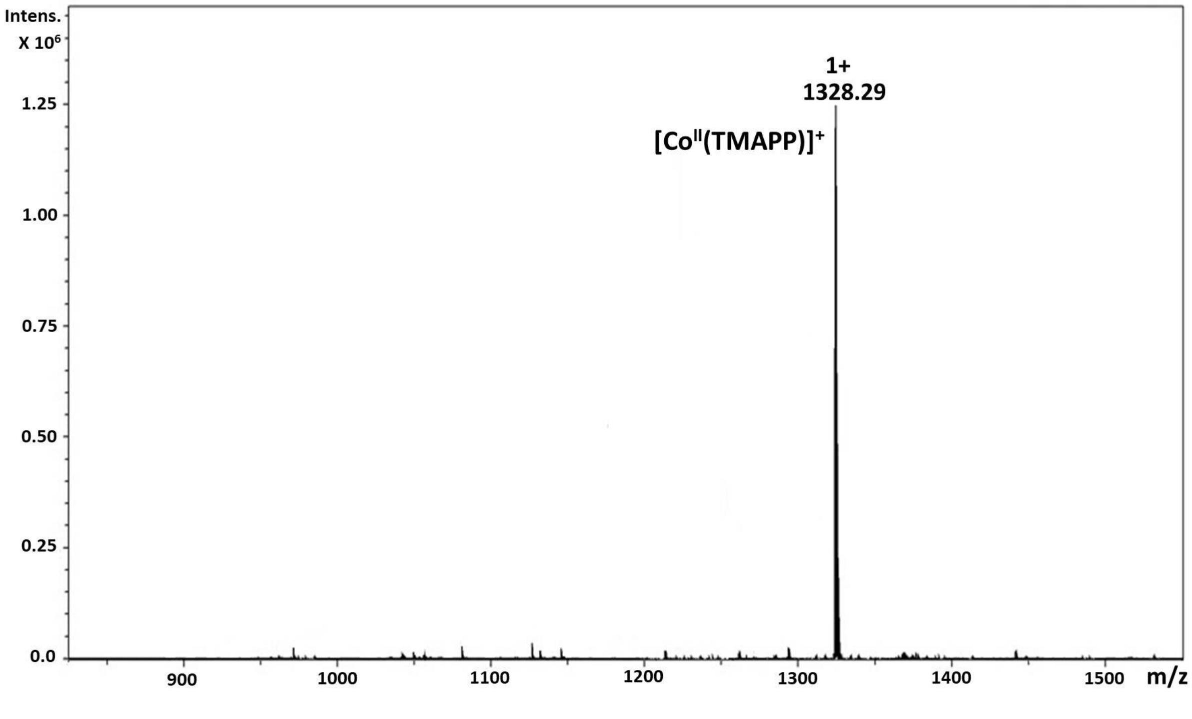

Fig. S6. Positive MALDI-TOF mass spectrum of [Co^{II}(TMAPP)] (**2**).

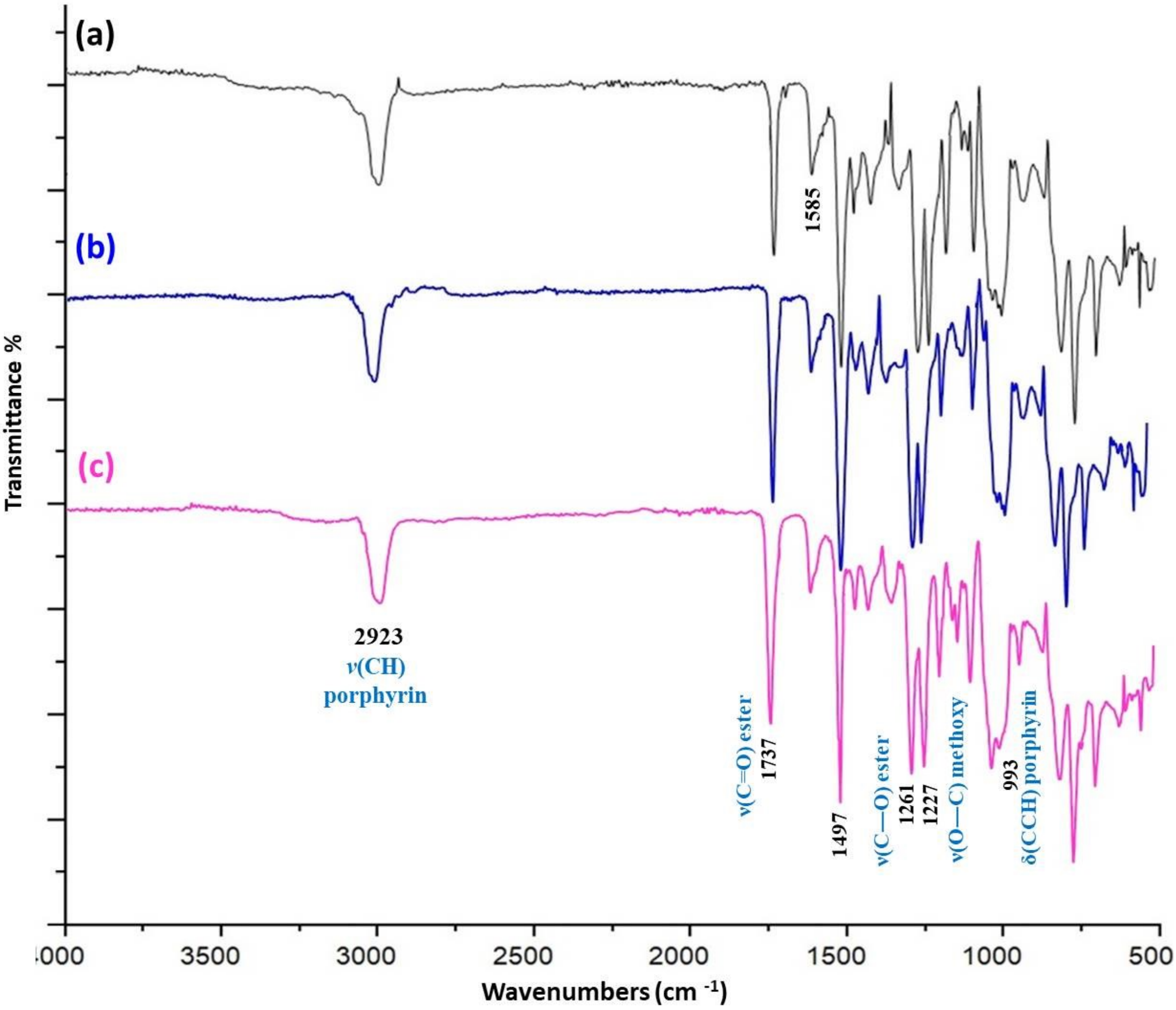

Fig. S6. FT-IR spectra of [Co^{II}(TMAPP)]: (a) before degradation, (b) after degradation of MB, and (c) after degradation of CV.

References

[1]. Z. Denden, K. Ezzayani, E. Saint-Aman, F. Loiseau, S. Najmudin, C. Bonifácio, J.-C. Daran and H. Nasri,. *Eur. J. Inorg. Chem*. 2015,**15**, 2596-2610.

[2]. N. Amiri, F. Ben Taheur, S. Chevreux, E. Wenger, G. Lemercier and H. Nasri, *Tetrahedron* 2017, **73**, 7011-7016.

[3]. N. Amiri, M. Hajji, F. Ben Taheur, S. Chevreux, T. Roisnel, G. Lemercier and H. Nasri, *J. Solid State Chem*. 2018, **258**, 477–484.

[4]. Y. X. Du, Z. Q. Zhang, Y. H. Yao and J. Li, *Inorg. Chem. Commun*. 2016, **64**, 19–22.

[5]. Y. Belghith, A. Mansour, J.-C. Daran and H. Nasri, *Open J Inorg Chem*. 2012, **2**, 81-87.

[6]. A. Mansour, Y. Belghith, M. S. Belkhiria, A. Bujaczb, V. Gue´rineau and H. Nasri, *J. Porphyrins Phthalocyanines* 2013, **17**, 1094–1103.

[7]. N. Amiri, S. Nouir, M. Hajji, T. Roisnel, T. Guerfel, G. Simonneaux and H. Nasri, *Saudi Chem. Soc*. 2019, 23, 781–794.

[8]. K. M. Kadish and M. M. Morrison, *J. Am. Chem. Soc*. 1976, **98**, 3326–3328.

[9]. M. Guergueb, S. Nasri, J. Brahmi, F. Loiseau, F. Molton, T. Roisnel, V. Guerineau, I. Turowska-Tyrk, K. Aouadi and Habib Nasri, *RSC Adv*. 2020,**10**, 6900–6918.

[10]. X. Ke, R. Kumar, M. Sankar and K. M. Kadish, *Inorg. Chem*. 2018, **57**, 1490–1503.

[11]. K. M. Kadish, X. H. Mu and X. Q. Lin, *Inorg. Chem*. 1988, **17**, 1489–1492.

[12]. L. A. Truxillo and D. G. Davis, *Anal. Chem*., 1975, **47**, 2260–2267.
